# Software-based analysis of bacteriophage genomes, physical ends, and packaging strategies

**DOI:** 10.1186/s12864-016-3018-2

**Published:** 2016-08-26

**Authors:** Bryan D. Merrill, Andy T. Ward, Julianne H. Grose, Sandra Hope

**Affiliations:** Department of Microbiology and Molecular Biology, Brigham Young University, 4007 LSB, Provo, UT 84602 USA

**Keywords:** Phage, Terminase, Phamerator, Phylogenetic tree, DNA packaging, Comparative genomics, Sequencing

## Abstract

**Background:**

Phage genome analysis is a rapidly growing field. Recurrent obstacles include software access and usability, as well as genome sequences that vary in sequence orientation and/or start position. Here we describe modifications to the phage comparative genomics software program, Phamerator, provide public access to the code, and include instructions for creating custom Phamerator databases. We further report genomic analysis techniques to determine phage packaging strategies and identification of the physical ends of phage genomes.

**Results:**

The original Phamerator code can be successfully modified and custom databases can be generated using the instructions we provide. Results of genome map comparisons within a custom database reveal obstacles in performing the comparisons if a published genome has an incorrect complementarity or an incorrect location of the first base of the genome, which are common issues in GenBank-downloaded sequence files. To address these issues, we review phage packaging strategies and provide results that demonstrate identification of the genome start location and orientation using raw sequencing data and software programs such as PAUSE and Consed to establish the location of the physical ends of the genome. These results include determination of exact direct terminal repeats (DTRs) or cohesive ends, or whether phages may use a headful packaging strategy. Phylogenetic analysis using ClustalO and phamily circles in Phamerator demonstrate that the large terminase gene can be used to identify the phage packaging strategy and thereby aide in identifying the physical ends of the genome.

**Conclusions:**

Using available online code, the Phamerator program can be customized and utilized to generate databases with individually selected genomes. These databases can then provide fruitful information in the comparative analysis of phages. Researchers can identify packaging strategies and physical ends of phage genomes using raw data from high-throughput sequencing in conjunction with phylogenetic analyses of large terminase proteins and the use of custom Phamerator databases. We promote publication of phage genomes in an orientation consistent with the physical structure of the phage chromosome and provide guidance for determining this structure.

**Electronic supplementary material:**

The online version of this article (doi:10.1186/s12864-016-3018-2) contains supplementary material, which is available to authorized users.

## Background

**Fig. 1:**
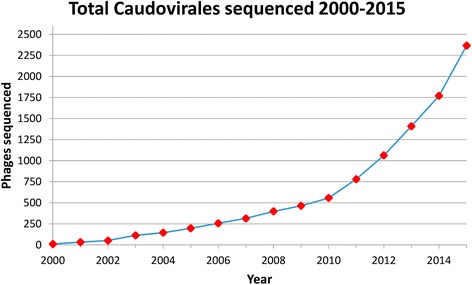
Total Caudovirales sequenced since 2000. This figure includes all complete genomes of Caudovirales sequenced and deposited in the “Nucleotide” NCBI database since 2000

Bacteriophages are the most abundant and diverse biological entities on earth [1]. Thousands of students and professors at hundreds of universities around the world are studying bacteriophages [2]. Low sequencing costs allow researchers to sequence and publish the genomes of phages they study. As a result, phage genomes are being added to GenBank at an exponential rate (Fig. 1).

Phamerator is a computer program [3] written to analyze the many Mycobacteriophages isolated and sequenced through the Howard Hughes Medical Institute (HHMI) Science Education Alliance-Phage Hunters Advancing Genomics and Evolutionary Science (SEA-PHAGES) program [2]. Phamerator is popular among the large groups studying Mycobacteriophages [4] and *Bacillus* phages [5, 6] and is steadily gaining traction in other areas of phage research [7–11].

Herein we describe software-based methods to study phage genomes, determine phage genome ends, and identify phage DNA packaging strategies. There are several limitations to the original version of Phamerator that we sought to overcome. First, as originally written, Phamerator could only read existing databases hosted on remote servers and could not create custom databases to be explored on local computers. Second, no detailed documentation existed to describe how to make custom databases or use features other than the graphical user interface. The goal of this work was to enhance the existing code, and to make Phamerator accessible to all phage researchers by providing instructions on how to build and use a custom database in Phamerator. In addition, we describe best practices when preparing phage genomes for publication and effective downstream analysis using Phamerator and other programs. These contributions enable phage researchers to use this powerful program and provide a basis for more consistent deposition of phage genomes into NCBI that will facilitate downstream analyses.

### Phamerator computer coding and database setup

Phamerator is written in Python and runs in the Linux Ubuntu operating system [3]. Ubuntu can be installed on any computer as a virtual machine through programs like VirtualBox (https://www.virtualbox.org). Phamerator compiles Structured Query Language (SQL) databases of bacteriophage genomes using GenBank [12] formatted files. Phamerator compares all gene products in the database using ClustalW [13] or ClustalO [14] and BLASTP [15] and then groups these gene products into “phamilies” (phams) based on percent identity or BLASTP expect value (E-value) with other gene products in the pham. Phamerator also prepares linear genome maps for gene order and content (genome synteny) comparison, and includes nucleotide homology output. Researchers can manually assign phages into different clusters within a database, such as groups based on genome similarity, [11, 16, 17], genera [18] or host preference [8].

Phamerator database setup requires four main processing steps. In the first and second steps, Phamerator aligns all possible pairs of gene products in the database using both BLASTP and ClustalW and saves all statistically significant results. In the third step, the user specifies an E-value and a percent identity used to group proteins into phamilies. Other versions of Phamerator have been modified to instead use kClust to assign phamilies [19, 20] and run natively on Windows, Linux, and MacOS. These phamilies can help identify homologous gene products [3]. In the final step, Phamerator identifies conserved domains in every protein in the database using the Conserved Domain Database (CDD) [21]. These tools provide powerful analyses to study gene synteny and conservation.

Phamerator reads the phage data stored in the SQL database and displays it in a graphical user interface. Phamerator has two main graphical outputs: linear genome maps and phamily circles. The features and purposes of these graphics are described in the original Phamerator publication [3].

### Main features of Phamerator for comparative phage genomics

**Fig. 2:**
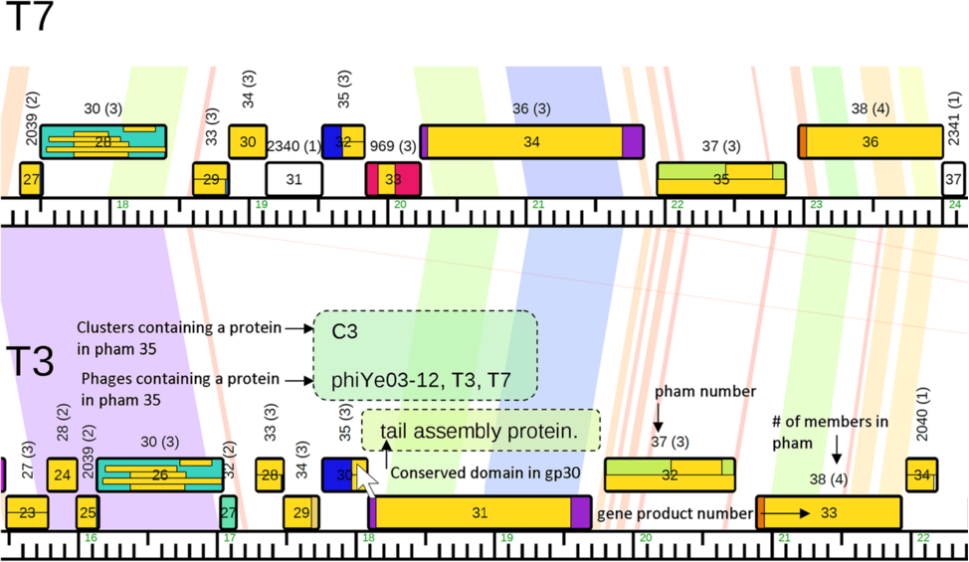
Phamerator genome map comparison. This linear genome map includes two similar phages published in a similar orientation. Colored lines connecting the genomes indicate the level of nucleotide similarity from purple (low E-value, high percent identity) to red (high E-value, lower percent identity). Horizontal yellow bars inside gene product boxes indicate conserved domains and represent the length of that domain relative to the length of the gene. When the mouse is hovered over one of the yellow conserved domains, a popup box will appear describing that domain (e.g., tail assembly protein, indicated by a dotted outline). When the mouse is hovered over a pham label, a popup box will appear (indicated by a dotted outline) which identifies the clusters and phages that contain a protein in a pham. Using these features, researchers can quickly identify conserved domains in any protein and which other phages in the database contain a homologous protein

Researchers can display a linear genome map of any number of phages in a database. The maps depict gene products as boxes that are colored by phamily or other parameters (Fig. 2). Colored lines connecting adjacent phage genomes indicate BLASTN homology from purple (low E-value, high percent identity) to red (high E-value, lower percent identity). These maps highlight mosaicism and synteny, and can be adjusted to align homologous genes or sections. Hovering the mouse over gene products will display text describing identified conserved domains and conservation of that gene product throughout phages in a user-defined cluster or throughout the whole database. Figure 2 demonstrates how a linear genome map containing phage genomes in a similar orientation can be used to identify homologous genes, conserved proteins, and conserved domains when compared to other phages in the database. Additional file 1 is a table that lists all of the phages included in the database used to generate the Phamerator figures in this paper.

**Fig. 3:**
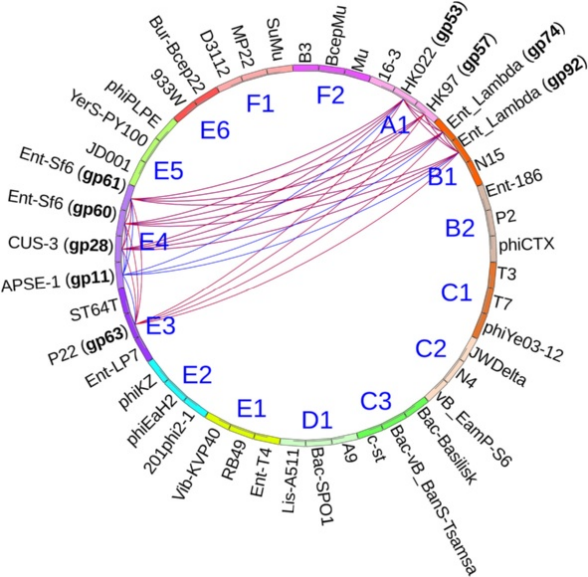
Phamily circle of pham 271, a Lambda family phage holin. This phamily circle displays the relationships of nine proteins that belong to pham 271. Conserved domains indicate these proteins are phage holins in the Lambda family. Cluster designations which reflect experimentally determined packaging strategies (see Additional file 1) are indicated inside the circle. Gene products connected by red lines are included in the pham because they have an E-value of less than 1e-50. Gene products connected by blue lines are included in the pham because they share more than 32.5 % identity

Phamerator also creates phamily circles for each gene product phamily (Fig. 3). These circles display phage names around the perimeter. Phages are organized around the circle according to user-defined cluster assignments. If a phage contains a gene product in the pham being displayed, the gene product number appears next the phage name. Inside the circle, lines connect proteins in the same pham based on ClustalW or BLASTP relationships. A blue line connecting two gene products indicates that they share greater than 32.5 % identity, a red line connecting two gene products indicates that they have an E-value of less than 1e-50 (Fig. 3). If the ClustalW and BLASTP parameters used to build phamilies vary, then lines may not be drawn if relationships fall below the default values of 32.5 % and 1e-50. Section 3.2, step 9 of Additional file 2 describes the process of building phamilies. We set ClustalW and BLASTP cutoff values for building protein phamilies in this database at 32.5 % and 1e-35, respectively. At this time, changing the parameters for building phamilies will not affect the parameters used to display pham circles.

**Fig. 4:**
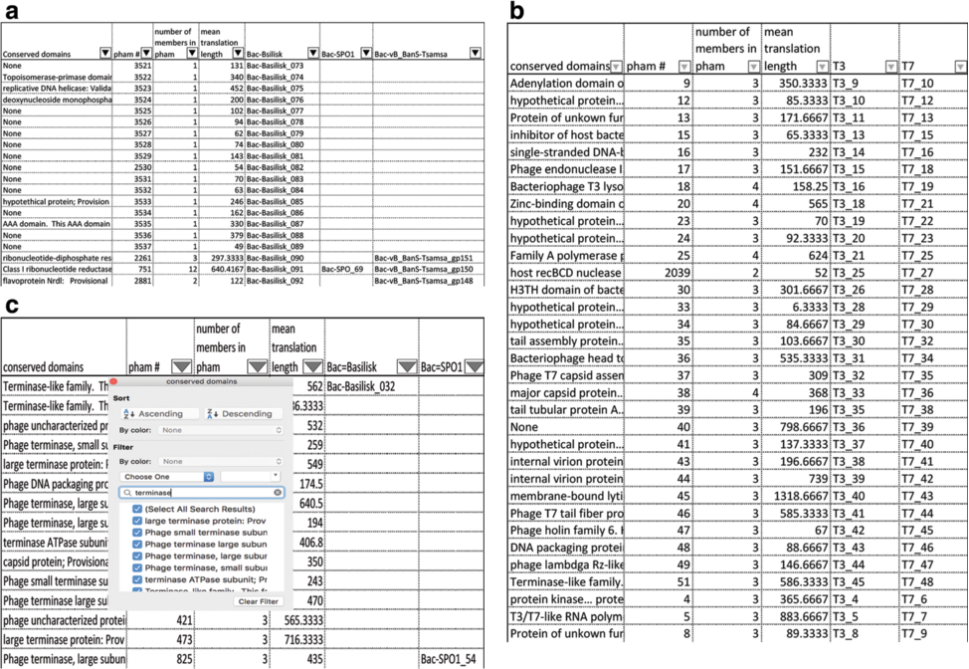
Excerpts of pham table exported from Phamerator. **a** The pham table is sorted by gene number in *Bacillus* phage Basilisk. Conserved domains and phamily members are identified for each gene. **b** Excerpts displaying only genes found in T3 and T7 (the T3/T7 conserved core genome). **c** A pham table filtered for conserved domains containing the word “terminase”. All phams containing gene products that are terminases are displayed

Phamerator exports two user-friendly spreadsheets: the “pham table” and the “cluster table”. The pham table lists phams down the left column and phage names across the top row. Gene products in each phage are placed on the row of the phamily they belong to. The cluster table also lists phams down the left column but lists the user-defined phage clusters across the top row. The number of gene products from each cluster that belong to each pham is listed. Each table lists conserved domains organized by phamily. Additional file 3 is a table that contains an example pham table and cluster table from a Phamerator database, while Fig. 4 contains excerpts from this table. By using sort and filter tools within a spreadsheet editor, these spreadsheets can be used to extract data including gene products common to a select group of phages, all gene products with an identified conserved domain, all members of the largest phamily, and more. Users can also quickly export custom sets of genomes, genes, or proteins.

### Phage genome orientation

Effective Phamerator analysis of similar phage genomes requires consistency in the genome orientation and the location of the first base. As phage genomes are published it is important that the orientation and complementarity are intentional, reflect physical properties of the phage chromosome, and are consistent with well-characterized phages. Phage genomes are currently deposited with a wide variety in the base one calls for even very similar phages [11]. Thus, one crucial step in preparing phage genomes from GenBank files for Phamerator and other analyses is to rearrange genomes that are oriented incorrectly so that genome content and gene order may be easily compared. Proper identification of physical ends and phage packaging strategies allows researchers to arrange phage genomes correctly before publishing them.

Although wet lab methods for determining phage ends and packaging strategies have been described previously [22], these experiments consume time and resources and may be inconclusive. Software-based methods using raw next-generation sequencing data provide insight into physical ends and packaging strategies [23]. These data can guide, clarify, or potentially replace wet lab experiments, especially when working with large datasets.

## Implementation

### Modifications to the original Phamerator code fixes errors and allows for continued compatibility

The original Phamerator code was retrieved and modified by the Brigham Young University (BYU) Life Sciences IT Department and the authors of this paper. The original Phamerator code is found at http://phamerator.csm.jmu.edu/files/phamerator.release/ and can be installed using Bazaar using instructions available at http://phagesdb.org. Our modifications to Phamerator allow local, custom databases to be easily created, altered, and viewed. These databases can contain both newly sequenced phage genomes and phage genomes retrieved from NCBI. The modified Phamerator code is deposited at http://github.com/byuphamerator/phamerator-dev/. A detailed list of changes is provided in Table 1.

**Table 1:** Phamerator Features and Modifications

Feature	Updates provided in new version	Justification
Biopython compatibility	Works with BioPython 1.64	Continued compatibility with future Biopython versions
Building the Phamerator database	Added prompts for username, password, server location, and database name at each step	The new prompts replace what was once written directly into the code
ClustalO alignments	ClustalO may be used instead of ClustalW to perform alignments	ClustalO is newer and is faster
Computation progress	Fixed script displaying the progress of BLAST and ClustalW	Helps users estimate when these jobs will finish
Pham and cluster tables	Column listing conserved domains for each pham was added to these tables	Used to quickly determine putative functions of proteins in a pham
Domain and pham labels in genome maps	Added whitespace to the right of these maps	Labels near the end of these maps are now visible
Delete BLAST and ClustalW scores	Users are prompted to delete or keep all scores when adding or removing phages	Scores can be deleted following major modifications to the database

This table describes features of Phamerator, the updates provided by the new version we provide, and the justification of why these modifications were necessary

### The Graphical User Interface (GUI) of Phamerator is run on various operating systems with the aid of virtual software

The graphical interface of Phamerator has wide usage among universities involved in the SEA-PHAGES program and is growing in popularity among other phage researchers as well. SEA-PHAGES members can download a pre-configured Ubuntu virtual hard drive file (www.hhmi.org/seawiki) and gain access to the Mycobacteriophage database managed by Graham Hatfull at the University of Pittsburg and Steve Cresawn at James Madison University. The virtual hard drive can be run using VirtualBox (www.virutalbox.org) or other virtualization software. At BYU, Phamerator is accessible in the Windows environment by forwarding an X11 window over SSH from a Linux virtual machine (VM) running on a server. This always-on VM keeps local computers fast as resources aren't spent running a local VM. This server VM allows multiple users on each VM, also saving users the time it takes to install and manage a virtual machine. North Carolina State University (NCSU) has also successfully built their own Phamerator databases which they currently use for teaching and research purposes. A Virtual Computing Lab at NCSU allows students to log on to a Ubuntu virtual machine from anywhere on campus and access Phamerator.

After a Phamerator database of phage genomes is compiled and processed it can be viewed and studied using the graphical user interface. Prior to our work, database setup was exclusive to the SEA-PHAGES program. The following section describes how to prepare a Phamerator database using GenBank-formatted genome sequences so that any user can prepare a custom database for analysis.

### A custom Phamerator database can be generated

Phamerator has three main parts: the graphical user interface (GUI), the Python scripts, and the SQL database. The GUI is the window used to view linear genome maps, pham circles, etc. Each Python script performs a specific function such as importing phages or computing Clustal scores. The SQL database is a set of linked tables where all of the phage gene sequences, alignment scores, etc. are stored. The database must be populated with phage genomes and processed before the end-user can view the desired genomes and access the features of Phamerator.

The following steps are used to create a Phamerator database containing user-specified phage genome sequences.

1. Install Ubuntu on a computer or inside a virtual machine.
2. Install Phamerator and the programs it needs to run.
3. Create a blank MYSQL database.
4. Insert table headers into the blank database so Phamerator knows where to store and access phage data.
5. Create GenBank-formatted files for recently sequenced phage genomes or retrieve phage GenBank files from NCBI. Use a program, such as DNA Master (http://cobamide2.bio.pitt.edu), to fix any formatting errors.
6. Import phage genome files into the SQL database.
7. Run Clustal comparisons on all phage gene products in the database. Each Clustal “job” compares one phage gene product against all others in the database and records significant alignments.
8. Run BLASTP comparisons on all phage gene products in the database. Each BLASTP “job” compares one phage gene product against all others and records significant E-values.
9. Run phamBuilder to group similar gene products into phamilies. Gene products are joined into a pham when they are similar to at least one other member by either a Clustal percent identity or BLASTP E-value at or above user-defined cutoffs. Commonly used values are 32.5 % identity and 1e-50 E-value [3].
10. Run cddSearch to identify conserved domains in gene products in the database using the CDD.
11. Export the database to a single SQL file to be shared with others.

Detailed instructions to execute these steps have been deposited at our website, http://phagehunters.byu.edu/Phamerator and are also included as Additional file 2. The instructions describe the process in detail to assist users through the technical tasks required to set up Phamerator. For example, Phamerator is currently only available for computers running Ubuntu. In most cases, this means that Ubuntu must be installed as a virtual machine. Processing a Phamerator database requires a computer with a powerful processor. An additional 40 GB of hard drive space is needed to set up a local copy of the CDD so conserved domains can be added to gene products in Phamerator. In the instruction manual, we provide descriptions of common errors that can occur due to variations in GenBank files and include a troubleshooting section for these errors. For example, GenBank files imported into Phamerator must contain unique locus tags, a “gene” feature, and a “CDS” feature for each gene. In addition, to avoid translation errors during importing, each gene in the file must use the “Bacterial and Plant Plastid” translation table. Furthermore, genomes that are arranged incorrectly or contain genes that wrap around the genome from the end to the beginning must first be modified using a program such as DNA Master, written by Dr. Jeffrey Lawrence and available online at http://cobamide2.bio.pitt.edu.

## Results and Discussion

### Publication of phage genomes without a standardized genome start location or orientation hinders analysis using comparative genomics software

**Fig. 5:**
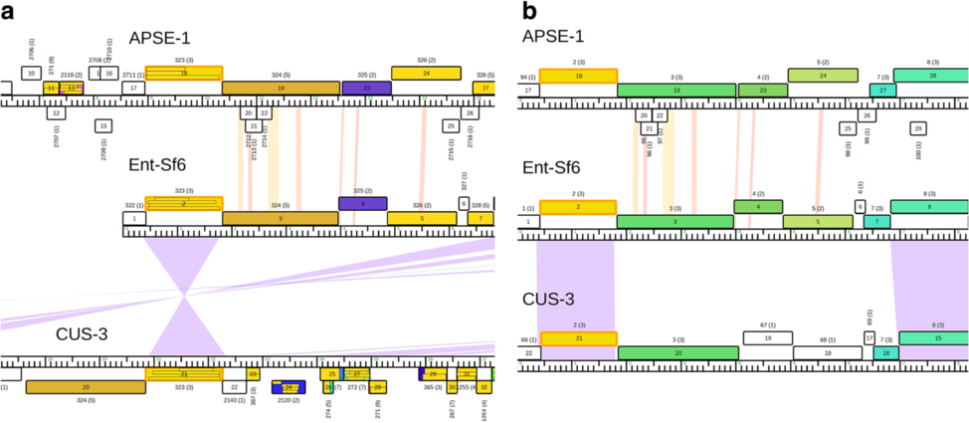
Linear genome map of three circularly permuted phages from the E4 cluster, which package chromosomes via the headful strategy. **a** Only Sf6 is arranged correctly. The large terminase protein is outlined in orange. Relative to phage SF6, APSE-1 and CUS-3 are arranged incorrectly and CUS-3 is also reverse-complemented. Lines connecting CUS-3 and SF6 indicate nucleotide homology. **b** Using DNA Master, APSE-1 and CUS-3 were rearranged and reversed complemented and these new files were reanalyzed using Phamerator for comparison. Original gene numbers were preserved

**Fig. 6:**
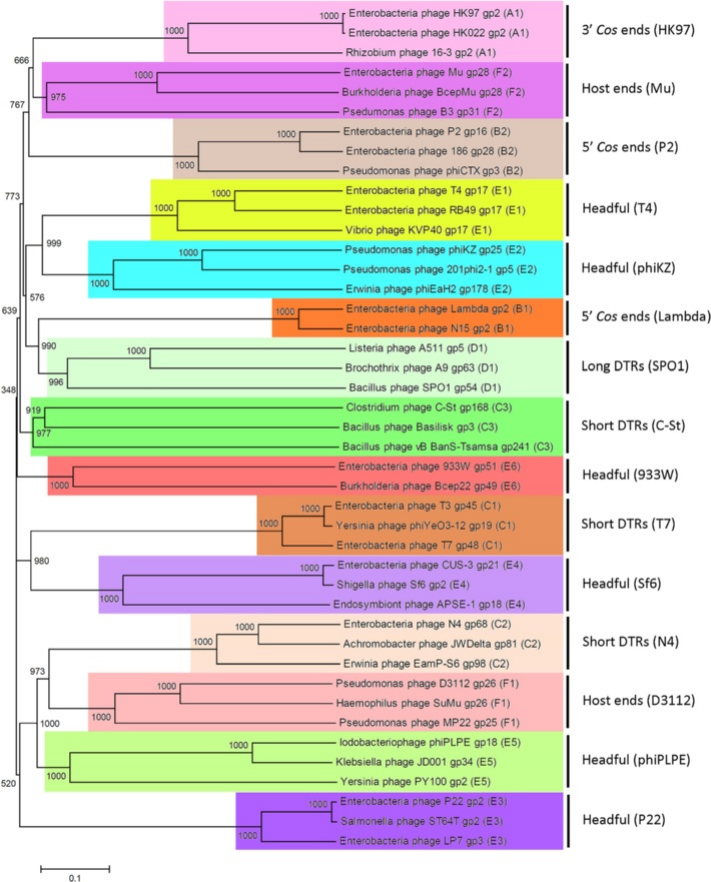
Neighbor-joining tree of large terminase proteins. This tree was generated by ClustalX [13], displayed in Mega6 [40], and contains large terminase sequences from phages with experimentally determined packaging mechanisms and physical ends (see Additional file 1). Bootstrap values are for 1000 trials. The scale bar shows 0.1 amino acid substitutions per site. We manually assigned clusters in Phamerator that correspond to packaging strategies. For example, phages that use 3’ *cos* ends (HK97) are assigned to cluster A1. This phylogenetic tree indicates that large terminase proteins sharing phamilies and packaging strategies also clade together

Similar phage genomes that begin near the same gene allow for easy identification and visualization of homologous regions using software such as Phamerator and other comparative analysis programs. When newly published genomes begin at a different gene or are reverse complemented relative to similar genomes, it becomes difficult to make direct comparisons. For example, Fig. 5a is a linear genome map of the three Sf6-like headful packaging phages as they are published on GenBank (E4 cluster, see Fig. 6). Phage Sf6 is oriented so that the terminase (gp2) is near the beginning of the genome in the forward direction. Although APSE-1 and CUS-3 are highly similar, they are not published in a similar orientation, making comparisons difficult. The terminases in APSE-1 and CUS-3 are gp18 and gp21, respectively. APSE-1 is published using the correct complementarity but the base one call is ~8.5 kb upstream relative to Sf6. The published genome of CUS-3 is reverse complemented relative to Sf6 and begins ~17.5 kb upstream (Fig. 5b). Although Phamerator can reverse complement genomes and align specific genes, it cannot assume a circular sequence and rearrange genomes to easily identify homology and synteny. Conflicting genome orientations is a problem not only with Phamerator, but is something that must be addressed before using other popular genome alignment comparisons such as MAUVE [24] or dot plot analysis programs. DNA Master is a program that can be used import GenBank files, rearrange genomes, and export FASTA or GenBank files (see instruction manual), but this can be time-consuming. We adduce a best practice to publish phage genomes in light of physical ends and packaging strategies and not based on artificial circularity or a previously published phage genome that may be oriented incorrectly. Accurate base one calls prior to publication will facilitate rapid, precise comparisons between similar phage genomes using Phamerator and many other programs. Prior to building a custom Phamerator database, we assess each phage genome to ensure consistency in the genome start position and orientation.

### Sequencing data can reveal phage DNA packaging strategy to select the genome start and orientation

Regardless of the packaging strategy or physical ends, all tailed bacteriophages (Caudovirales) end up with a linear DNA molecule packaged in the capsid of the mature virion [22]. This genome is then injected into a new host, wherein most phage chromosomes circularize. The mechanism of circularization is dependent on the packaging strategy and the type of physical ends produced. Therefore, identification of the packaging strategy can reveal the location of the physical start of a phage genome, and sequencing data can often be analyzed to determine the packaging strategy used [23, 25, 26].

**Fig. 7:**
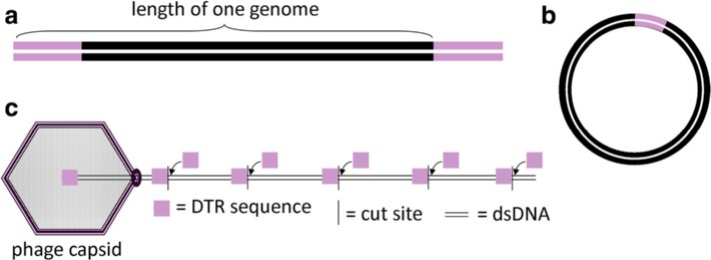
Physical structure, circularization, and packaging mechanism of a phage with exact direct terminal repeats (DTR) at each end. **a** The DNA inside the phage virion before infection has the same sequence at both ends. These ends are identical in each virion. **b** After infection, the ends undergo homologous recombination to form a circular DNA molecule. **c** A linear concatemer is generated via rolling circle replication. The repeated ends are duplicated while the DNA is being packaged. Each virion has identical repeats at each end

Bacteriophages that use homologous recombination to generate circular chromosomes following infection must have an identical sequence at each end of the linear chromosome (Fig. 7a). Some phages use exact direct terminal repeats (DTRs) to accomplish this. These repeats can be short (200–700 bp) or long (up to 16 kbp). Following homologous recombination, the circular chromosome contains exactly one copy of the DTR (b). The circular chromosome is replicated via theta and sigma (or rolling circle) replication, forming linear concatemers. The concatemers contain only one copy of the repeat sequence between each genome-length (Fig. 7c). The repeat between the next genome-length and the one being packaged are duplicated so that each virion receives a chromosome with an identical repeat at each end [27–29]. The raw data for these sequences indicate that twice as many reads cover the exact DTR when compared with the rest of the genome since the exact DTR is found twice in each phage chromosome. Thus, a phage likely has exact DTRs if it has an area where the number of reads mapped to the consensus suddenly doubles relative to the surrounding sequence.

**Fig. 8:**
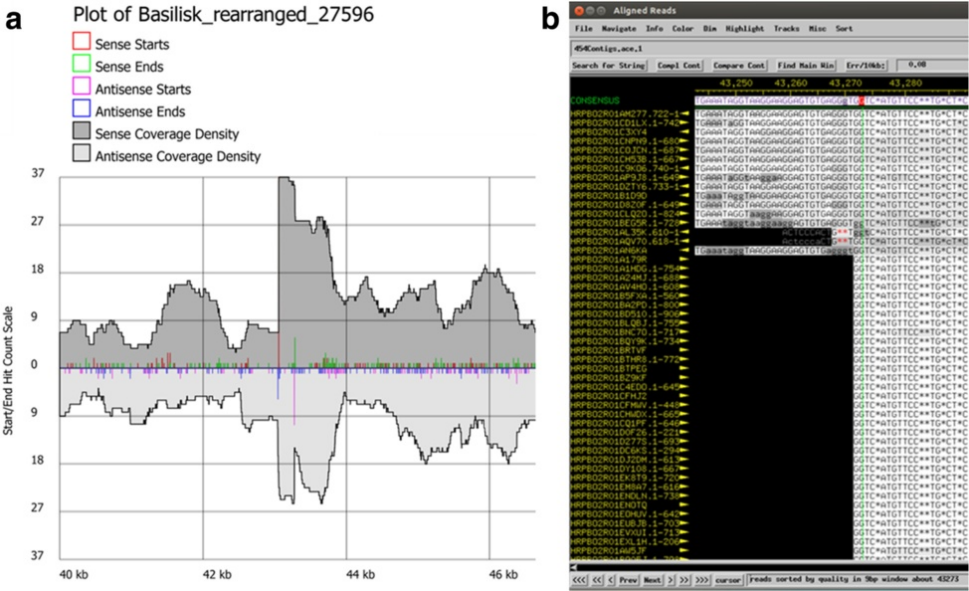
Analysis of exact DTRs in *Bacillus* phage Basilisk. **a** PAUSE analysis graphs the number of reads mapped to the Basilisk genome. The region between the sense and antisense starts and ends indicates the location of the short exact DTR in *Bacillus* phage Basilisk, which was used to call base one [6]. **b** Consed shows a sharp increase in coverage near the left end (sense start) of the exact DTR in *Bacillus* phage Basilisk. This location corresponds to the sense start which is marked by a tall read spike in Fig. 8a

The Pile-up Analysis Using Starts & Ends (PAUSE) program (https://cpt.tamu.edu/computer-resources/pause) looks for DTRs based on changes in coverage depth in reads that are aligned to the assembled phage genome and predicts the sequence and length of exact DTRs. PAUSE takes two inputs: (1) the finished FASTA file containing a phage genome and (2) the raw sequencing data in SFF or FASTQ format. Instructions for PAUSE are available at https://cpt.tamu.edu/analysis-with-pause/. PAUSE returns a plot of genome length versus coverage and predicts where DTR sequences begin and end (Fig. 8a). Consed [30] or another genome viewer can show individual reads mapped to the genome to visualize these changes in coverage. The beginning and end of DTR sequences are marked by sharp increases or drops in fold coverage (Fig. 8b). Each phage genome can be scrutinized to see if it contains repeats. If so, the sequence can be oriented true to the phage chromosome it represents with a repeat region on each end and the genome in the middle.

**Fig. 9:**
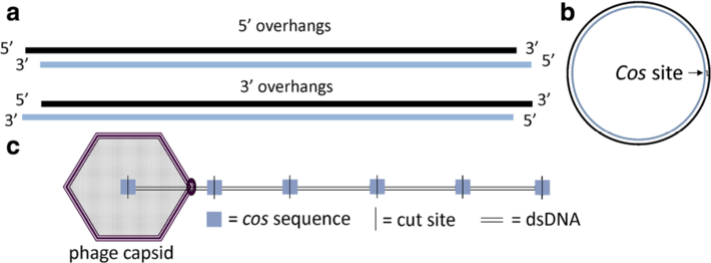
Physical structure, circularization, and packaging mechanism of a phage with cohesive ends. **a** Structure of DNA inside phage virion before infection. Phages with cohesive ends can have 3’ or 5’ overhangs. **b** Shortly after infection, the sticky ends are ligated. The chromosome is replicated via rolling circle replication during the lytic phase. **c** Exactly one genome length is packaged into each phage capsid. The terminase protein cuts at the *cos* site, leaving 5’ or 3’ overhangs

If no exact DTRs are identifiable, the phage may have cohesive ends or may be circularly permuted due to headful packaging. Phages with cohesive ends have a 3’ or 5’ overhang on each end of the phage chromosome (Fig. 9a). Before the chromosome is replicated, complementary overhangs will base-pair and the DNA is ligated into a circular molecule (Fig. 9b). A polymerase travels around the circular chromosome and produces linear concatemers up to ten genomes in length [31]. Overhangs are created when the large terminase identifies a specific *cos* site in the concatemer, starts packaging the DNA, and cleaves the DNA when the next *cos* site appears (Fig. 9c). The terminase cuts precisely at the *cos* site each time and packaging occurs with exactly one genome-length sequence in each phage capsid.

**Fig. 10:**
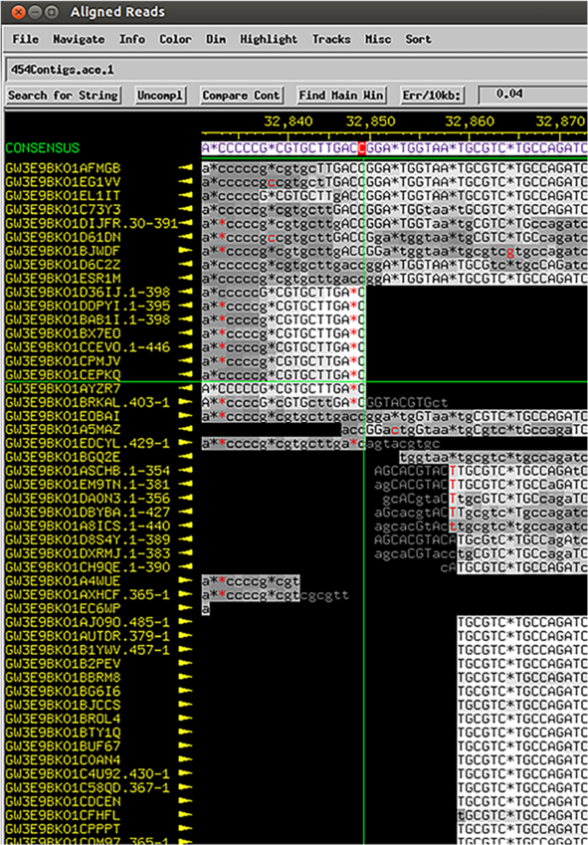
Consed visualization of *cos* overhang sequence. Consed shows a sharp drop in coverage over the 3’ overhang in *Mycobacterium* phage Atkinbua

**Fig. 11:**
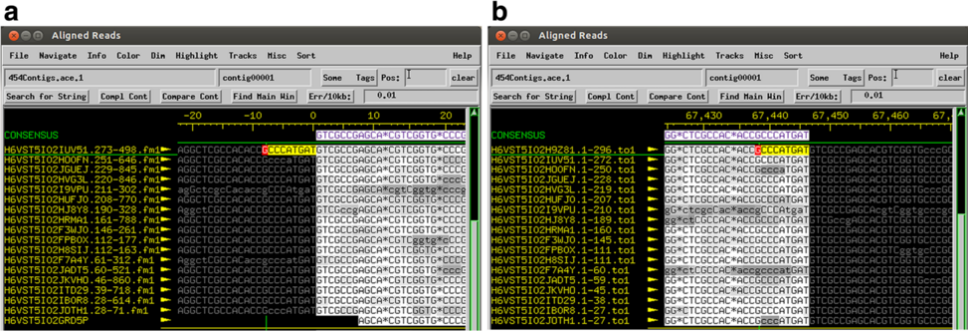
Consed visualization of wrap-around reads. The assembled contig for *Mycobacterium* phage Girly (http://phagesdb.org) contains reads that wrap around the ends of genome. The highlighted sequence to the left of the genome start **a** is the same as the last few base pairs at the end of the genome **b**

Phages with cohesive ends occasionally produce a distinctive pattern in read coverage at the *cos* site. To identify an area to search for this pattern, we first determine the location of the large terminase gene using BLASTX and an Entrez query of “terminase.” The *cos* site is often near the terminase genes. Chromosomes of phages with cohesive ends do not contain any repeated elements like phages that have exact DTRs or are circularly permuted and may or may not generate an artificially circular sequence. However, the ends of a few sequenced chromosomes can be ligated together producing reads that go from one end of the genome to the other. This relatively rare ligation event results in a sudden drop in fold coverage over the precise location of the *cos* site (4–19 base pairs) (Fig. 10). The lower-coverage *cos* site will also be flanked by many reads that begin or end at an identical location immediately flanking the *cos* site. If this coverage drop at the *cos* site is identifiable, the ends of the phage genome in Consed will show reads that run off one end of the genome and coincide with bases at the other end if the genome is complete (Fig. 11). If the genome ends don’t show any reads wrapping around, it is likely that the cohesive ends were not sequenced. In this case, the returned assembly likely spans one cohesive end to the other and does not actually include the overhang sequence on either end. At this point, it is possible to design PCR primers that will identify the sequence of these ends [2].

**Fig. 12:**
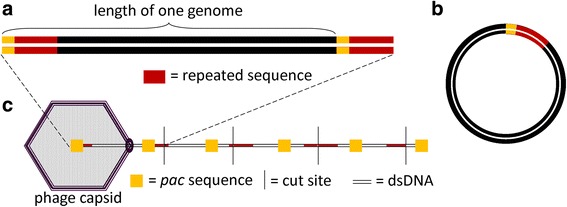
Physical structure, circularization, and packaging mechanism of a phage that uses headful packaging. **a** This figure represents the first phage chromosome packaged from a linear concatemer. The DNA inside the phage virion before infection has a similar DNA sequence at both ends. The repeat sequences at the ends of each chromosome vary from phage to phage. The bracket indicates exactly one genome-length (from one *pac* sequence to the next). **b** After infection, the ends undergo homologous recombination to form a circular DNA molecule that contains exactly one genome-length and one *pac* site. A linear concatemer is generated via rolling circle replication. **c** Beginning at the *pac* site, the terminase inserts the DNA into the capsid. The terminase creates imprecise cuts after slightly more than one genome length is packaged into the capsid, generating a repeated sequence at each end. Thus, the position of the *pac* site varies in each subsequent virion

The chromosomes of free virions that use headful packaging have a direct terminal repeat sequence on each end but these sequences vary among progeny phages; i.e., these repeats are not exact (Fig. 12a). The phage chromosomes circularize using homologous recombination (Fig. 12b) and form linear concatemers following replication. The terminase protein recognizes a specific site on the DNA called the *pac* sequence. The terminase cuts at or often near the *pac* site and begins inserting the first genome-length of the concatemer into a capsid until the capsid is full (Fig. 12c) and packages more than one genome-length (102–110 %) into each capsid. Unlike phages with exact DTRs that package the exact same sequence in each virion, phages that use headful packaging are unlikely to produce two virions that have the same sequence length starting and ending at the same location. Because slightly more than a genome-length is packaged, the first DNA base packaged in a given capsid can theoretically be any base in the genome and progeny virion chromosomes are circularly permuted.

Phages that have circularly permuted DTRs due to headful packaging will always show reads that run off one end of the genome when sequenced completely. These wrap-around reads contain bases coinciding with the other end of the genome (Fig. 11). If PAUSE shows consistent read depth throughout the genome, wrap-around reads are identified by Consed, no putative exact DTR repeat regions are identified, and there are no sudden drops in coverage near the large terminase gene indicative of cohesive ends (Fig. 10), then the phage is likely circularly permuted and uses headful packaging.

Phages rely on terminase proteins to identify replicated phage chromosomes from among the other DNA inside of the host. Terminases package phage chromosomes into phage capsids and cut concatemers into genome-sized lengths. The role of the terminase varies depending on the packaging mechanism. Therefore, terminases with similar amino acid sequences usually package DNA using similar mechanisms and create similar physical ends [22, 23]. Phylogenetic analysis has been used to gain additional insight into the packaging strategies of novel or poorly-studied phages [32–34] and is one way to predict the type of ends, including whether a phage has host ends. Analysis of large terminase proteins from phages listed in Additional file 1 indicate that large terminases with similar packaging strategies tend to clade together (Fig. 6). The clades of the phylogenetic tree correspond exactly to the cluster grouping that was assigned in Phamerator based on the Phams to which each large terminase belongs (A1-F2). Casjens and Gilcrease reported packaging strategies based on phylogenetic analysis and defined 11 groups: 5’ cos (Lambda, P2); 3’ cos (HK97), headful (P2, Sf6, T4, 933 W, GTA), host ends (Mu and D3112), and short DTRs (T7) [22]. Here, we propose five additional groups based on phylogenetic and Phamerator analysis: short DTRs (N4, C-st); headful (phiPLPE, phiKZ); and long DTRs (SPO1).

There are several considerations in making a phylogenetic tree containing large terminases. Although large terminases are well-conserved and are even similar among phages that infect different hosts, the overall diversity of large terminases is often too great to reliably analyze them all in one phylogenetic tree. This diversity causes instability of the branches and nodes as additional sequences are added. When adding a large terminase protein to a phylogenetic tree, some stability can be maintained by also including several BLAST hits that are similar to the terminase being queried, especially those hits that come from phages with experimentally determined packaging strategies.

Read pileups, wrap-around reads, changes in coverage density, and terminase phylogenies can guide researchers in making the appropriate “base one” call prior to publication or in designing wet lab experiments to verify the phage ends and packaging strategies. Exact DTRs in phages can be annotated [35] and these genomes are generally published with one repeat sequence on each end [6].

The complementarity of the genome is considered when making a base one call for phages that have exact DTRs, have host ends, or use protein-primed replication. For phages with cohesive ends, 5’ overhangs are placed at the beginning of the published genome, and 3’ overhangs are placed at the end. Base one calls for circularly permuted phages are more complicated because software-based methods cannot yet identify the *pac* sequence or *pac* fragment by looking at changes in coverage. Wet lab methods can occasionally identify the *pac* fragment as a piece of DNA that spans between the origin of replication and the site where the terminase makes the first cut. Because the large terminase protein is responsible for identifying and cutting at the *pac* site, the sequence of the *pac* site and the sequence of the large terminase protein often lie very close to each other, with the *pac* site often just upstream of the large terminase protein [22]. We typically determine base one calls in circularly permuted phages at or just upstream of the large terminase gene with the large terminase gene in the forward direction. Standardizing base one call methods for all phage types, especially for circularly permuted phages, will facilitate comparison of phage genomes and easier identification of homologs.

Although the analyses we describe of high-throughput data can give a good indication of the packaging strategy and the physical ends of the phage chromosome, the data may not always provide a definitive answer. For instance, at least two packaging mechanisms are known produce linear chromosomes with no wrap-around sequences, exemplified by phage Mu and phage phi29. Such packaging strategies may be difficult to distinguish from phages with cohesive ends that do not generate artificially circular sequences. Phage Mu inserts copies of its DNA into the host chromosome via replicative transposition [36]. When Mu DNA is excised from the host chromosome prior to being packaged, segments of the host chromosome become the ends of the linear phage DNA. Each segment of DNA packaged into a progeny phage contains different ends since they all came from different parts of the bacterial chromosome. These chromosomes are circularized [37] but are not believed to produce artificially circular genomes when sequenced. Phages like *Bacillus* phage phi29 also circularize in the host but have a protein covalently linked to each end that serves to prime DNA replication [38]. Phages with host ends or terminal proteins do not generate artificially circular sequences because there is no repeated sequence at the phage ends. Raw sequencing data may rule out cohesive ends, headful packaging, and exact DTRs without confirming whether a phage has host ends or covalent terminal proteins. Wet lab experiments, similarity to previously sequenced and characterized phages, or comparison of large terminase proteins are necessary to verify whether phages have host ends or covalent terminal proteins [22].

### A custom Phamerator database can be used to identify packaging strategies based on the large terminase protein

**Fig. 13:**
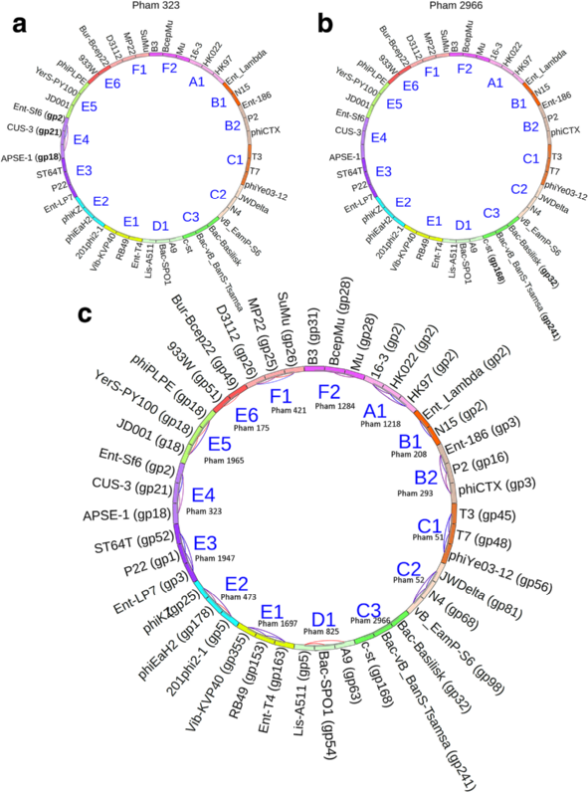
Phamily circles indicate relationships of large terminase proteins. Clusters (A1-F2) were intentionally set to group phages with similar packaging strategies together. **a** Pham 323 contains only three large terminase proteins, indicated by bolded gp designations. The three phages that encode these terminases belong to cluster E4, which includes phages that use headful packaging (Sf6) [41, 42]. **b** Pham 2966 contains only three large terminases, indicated by bolded gp designations. The three phages that contain these terminases belong to cluster C3, which includes phages that have short exact DTRs (C-st). These proteins meet the cutoff parameters to be included in pham 2966, but do not meet the parameters required to draw connecting lines (see Fig. 13a). **c** An overlay of 15 pham circles represents large terminase proteins for every phage in the database. This circle indicates that large terminases grouped into the same pham belong to phages that use the same packaging strategy. In this database, no terminases were grouped with terminases belonging to phages that use a different packaging strategy. Gene products connected by red lines have an E-value of less than 1e-50. Gene products connected by blue lines share more than 32.5 % identity

Using Phamerator, phamily circles (introduced in Fig. 3) can be created for each phamily in the database and are also a useful tool for identifying packaging strategies. As discussed above, gene products are included in phamilies if they have a sufficiently high E-value or percent identity with at least one other gene product in the phamily. If the requirements for inclusion in a pham are stringent (similar to the default parameters), two terminases in the same pham likely use the same packaging strategy. If a particular phage contains a gene product belonging to the pham, then the gene number is listed next to the phage name (Fig. 13). As described previously, ClustalW and BLASTP relationships are indicated by connecting blue and red lines, respectively (Fig. 13a), except where phamily building parameters fall below 32.5 % and 1e-50. In this case, gene product numbers will be listed next to proteins in a pham but no connecting lines will be drawn (see Fig. 13b). In this database, the large terminases of phages using the same packaging strategy grouped into phamilies that contained no other members as represented by the lack of any line connecting phages of different clusters (clusters were intentionally pre-assigned by packaging strategy). Figure 13c depicts the relationships of all phamilies containing large terminase proteins by overlaying 15 phamily circles generated by Phamerator on top of each other (the Phamerator database used in this analysis can be downloaded from http://phagehunters.byu.edu/Phamerator).

## Conclusions

Our modifications to Phamerator combined with new documentation for setting up custom databases and troubleshooting errors make this powerful software widely available and user-friendly. We plan to release additional updates to Phamerator that will add new features and resolve persistent problems, including: display of pham circle relationships using parameters identical to those used to build phamilies, display of pham tooltips when the map alignment is changed, display of pham circles when no phages are assigned to the singleton cluster, and display of phage tRNAs on the linear genome map.

Using the techniques we described, high-throughput sequencing data can be used to determine packaging strategies and physical ends of phage chromosomes. Understanding the principles of phage genome packaging and utilizing phage genome comparison software will lead to informed decisions when publishing phage genomes, standardizing phage genome submission. Because phage genomes are being added to GenBank at a rapid rate, publishing them in a consistent manner will allow straightforward phage characterization and comparison using Phamerator and other programs.

## Methods

Accession numbers for the 43 phage genomes and large terminase proteins used in this paper are listed in Additional file 1. We downloaded bacteriophage genomes in GenBank format from NCBI and used them to build a Phamerator database according to the instructions found in Additional file 2. Phage gene products in these genomes were compiled into a pham if they shared a BLASTP E-value of 1e-35 or less or 32.5 % identity as computed by ClustalO with at least one other gene product in the pham. The phylogenetic tree of 43 large terminase proteins was computed using the neighbor-joining method using ClustalX [13] with a bootstrap value 1000 and was displayed using Dendroscope [39].

## Additional files

Additional file 1: Table of phages and large terminase proteins used in Phamerator database. (XLSX 15 kb) Additional file 2: Phamerator instructions. (PDF 820 kb) Additional file 3: Pham table and cluster table generated by Phamerator. (XLSX 774 kb)
