# Supplementary material for: Software-based analysis of bacteriophage genomes, physical ends, and packaging strategies
Source: BMC Genomics. 2016 Aug 26;17(1):679. doi: 10.1186/s12864-016-3018-2 (PMC5000459; doi:10.1186/s12864-016-3018-2)
Supplement: Additional file 2: — Phamerator instructions. (PDF 820 kb) [file 12864_2016_3018_MOESM2_ESM.pdf]

Setting Up Phamerator and Creating Usable Databases for Bacteriophage Comparative Genomics

Bryan D. Merrill, Andy T. Ward, Julianne H. Grose, Sandra H. Burnett

Table of Contents

I. INTRODUCTION

II. HARDWARE REQUIREMENTS

III. HOW TO SET UP A UBUNTU VIRTUAL MACHINE
1. Download Ubuntu 14.04 LTS
2. Install the latest version of VirtualBox (or other virtualization software) and create a virtual hard drive
3. Install Ubuntu on your new virtual hard drive
4. Install the VirtualBox extensions package (Guest Additions)
5. Install Ubuntu Updates
6. Learn to use basic Terminal commands

IV. HOW TO INSTALL PHAMERATOR AND OTHER NECESSARY PROGRAMS
1. Use sudo apt-get to install software from the Internet
2. Start Phamerator and download BLAST

V. HOW TO BUILD A PHAMERATOR DATABASE
1. Prepare a new SQL database
2. Collect GenBank files and determine if they are ready to import into Phamerator
3. Using DNA Master to prepare GenBank files from NCBI for Phamerator
4. Using DNA Master to import FASTA files, auto-annotate them, and generate GenBank files for Phamerator
5. Using DNA Master to create a GenBank file
6. Import GenBank “fixed” files into Phamerator
7. Adding cluster designations to the Phamerator database
8. Starting Phamerator
9. Perform ClustalW computations on the Phamerator database
10. Perform BLAST computations on the Phamerator database
11. Check the progress of ClustalW or BLAST
12. Assign phamilies (phams) for gene products in the Phamerator database
13. Setting up a local copy of the Conserved Domain Database (CDD)
14. Using the Conserved Domain Database (CDD) Search
15. Modifying the Phamerator Database

VI. HOW TO USE THE PHAMERATOR USER INTERFACE TO EXPLORE THE DATABASE
1. Displaying linear genome maps
2. Displaying phamily circles
3. Exporting Pham and Cluster Tables
4. Exporting genomes, genes, or proteomes
5. Accessing the Phamerator Quick Start User Manual

VII. CONCLUSION

VIII. TROUBLESHOOTING AND FREQUENTLY ASKED QUESTIONS

IX. APPENDIX OF PHAMERATOR TERMINAL COMMANDS

I. INTRODUCTION

Phamerator was written by Dr. Steven Cresawn to analyze Mycobacteriophages that were isolated and sequenced as part of the HHMI SEA-PHAGES project headed by Dr. Graham Hatfull. As proof of its utility, Phamerator is currently being used by hundreds of research labs to analyze many types of phages. Phamerator is a compilation of Python scripts that runs in Linux Ubuntu. Different scripts import GenBank formatted phage genomes into an SQL database, perform BLAST and ClustalW computations, group similar gene products into phamilies, and identify conserved protein domains using the Conserved Domain Database (CDD). Each of these scripts records the results in the SQL database. The user interface reads the database, displays information, and has many forms of data output including custom sets of genomes, proteomes, linear genome map comparisons, phamily circles, and tables.

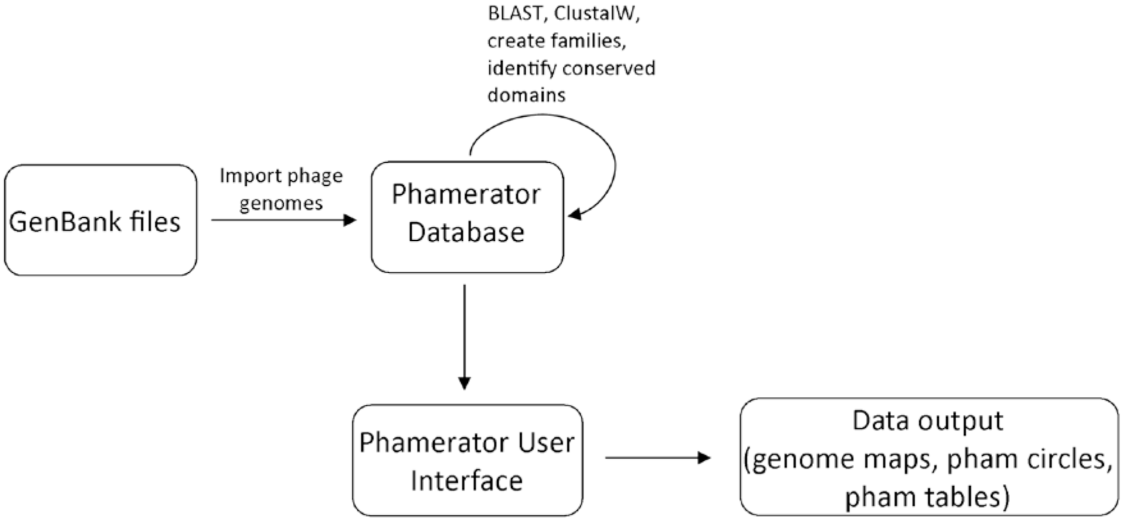

To install Phamerator and set up the database, users need to use Terminal, the Ubuntu command-line interface called Terminal. These instructions will describe how to set up Ubuntu, install Phamerator and the other essential programs, set up the SQL database, import prepared phage GenBank sequence files, perform computations on the database, and use the Phamerator graphical user interface to explore your database.

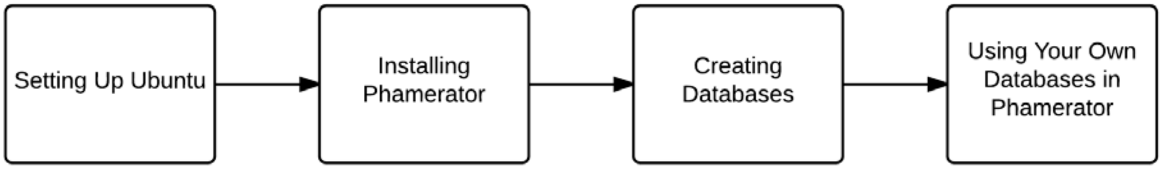

II. <u>HARDWARE REQUIREMENTS</u>

There are several requirements to setting up Phamerator. These instructions will work for a computer running Windows, Mac OS X, or a computer running Ubuntu natively.
To explore Phamerator databases you will need a computer that has:

- at least 20 GB free on the hard drive
- at least 4 GB of RAM
- at least a dual core processor

Building Phamerator databases requires significantly more hard drive space and computing power. We recommend a computer with:

- a 64-bit operating system
- an i7 processor (or equivalent)
- at least 8 GB of RAM

The time needed to process a database increases exponentially with each phage added to it. If adding conserved domain data to Phamerator databases, 100 GB of free hard drive space is recommended.

III. <u>HOW TO SET UP A UBUNTU VIRTUAL MACHINE</u>

Skip this section if the computer is running Ubuntu natively.

1. <u>Download Ubuntu 14.04 LTS</u>
   a. Visit the link http://www.ubuntu.com/download/desktop/
   b. Download 64-bit Ubuntu (the top option)

2. <u>Install the latest version of VirtualBox (or other virtualization software) and create a virtual hard drive</u>

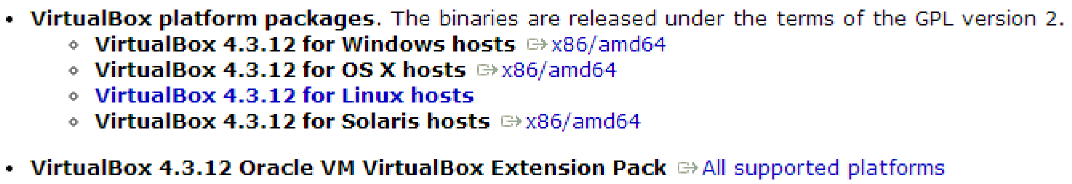

Figure 1 – Screenshot from VirtualBox download page

   a. Download the latest version of Oracle VirtualBox for your operating system, as well as the universal extension pack from the following website. https://www.virtualbox.org/wiki/Downloads (see Figure 1)
   b. Install VirtualBox and the extension pack you downloaded in step 2a, then start VirtualBox.
   c. After VirtualBox opens, Click the blue circular icon that says "New." Type a name for your new virtual machine (such as "Ubuntu Phamerator"). Choose "Linux" and "Ubuntu (64-bit)". Click "Next." See Figure 2.

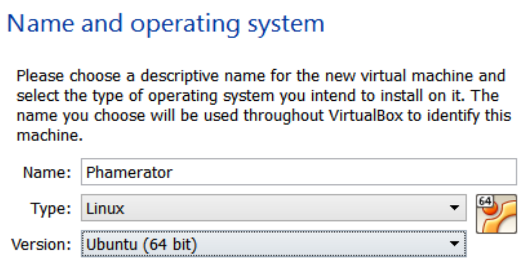

Figure 2 – Name and operating system of new Virtual Machine

   d. Choose how much RAM your virtual machine will have access to. Give it up to half of what is installed in the computer. Click "Next."
   e. Choose "Create new virtual hard drive now", and click "Create." In the window that pops up, choose "VDMK (Virtual Machine Disk), and click "Next."
   f. Choose "Fixed Size," and click "Next."
   g. Don't change the name of the virtual hard drive file, but change the size of the virtual hard drive. About 20.00 GB will be sufficient if you are only viewing databases or are not adding conserved domains. Otherwise, choose 80.00 GB . Click "Create."

h. To allocate more processor cores to your new virtual machine, choose "Settings," then "System," then the Processors tab.

i. To enable 3D acceleration, check that option under the "Display" tab.

3. <u>Install Ubuntu on your new virtual hard drive</u>

a. Start VirtualBox (if it is not running), click once on your new virtual machine, and click the "Settings" button at the top. A new window will open with options on the left like you see in Figure 3.

b. Click "Storage." Click on "Empty" next to the CD logo under Controller: IDE, then click the CD logo on the right (shown by red arrow in Figure 4). Choose "Choose virtual CD/DVD disk file" and navigate to the Ubuntu .iso file called "ubuntu-……-desktop-…..iso". Click "Open." Click "Ok."

c. Click on your virtual machine, and click the "Start" button with the green arrow.

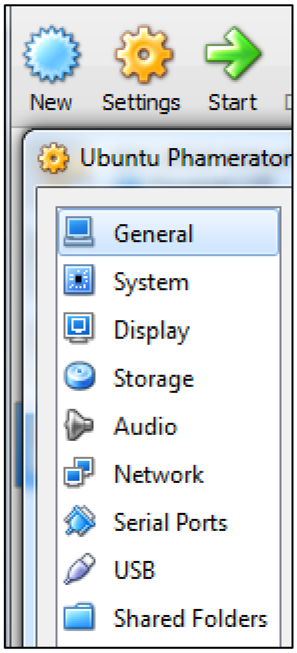

Figure 3 – Options found in "Settings" menu.

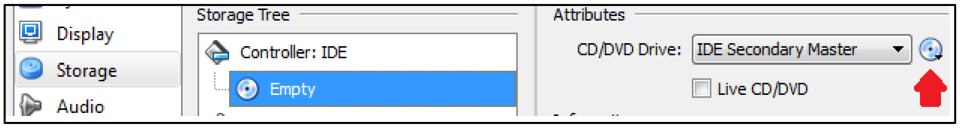

Figure 4 – Adding the .iso file to the Ubuntu virtual machine

d. A new window will pop up, and after a few seconds you will see a list of languages on the left, an option that says "Try Ubuntu" and an option that says "Install Ubuntu." Click on "Install Ubuntu."

e. In the next window, click the checkboxes that say "Download updates while installing" and "Install this third-party software." Click "Continue."

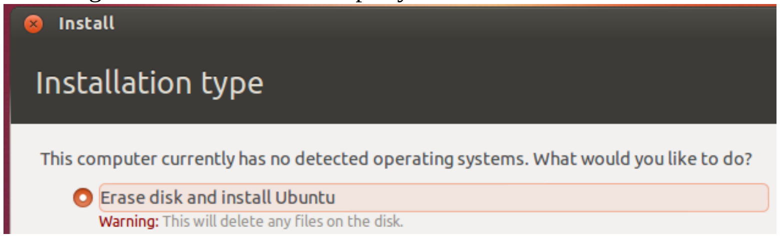

Figure 5 – Erase disk and install Ubuntu

f. The next step will look like Figure 5. Choose "Erase disk and install Ubuntu" and click "Continue."

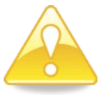

WARNING – This will NOT erase the hard drive on your computer to install Ubuntu. You are only installing Ubuntu inside the "virtual hard drive" you created, which is really just a file. You will NOT lose any of your data by choosing this option.

g. As Ubuntu is installing, you can enter information about your computer.

i. Enter your location by typing in your city, state, and country.

ii. Choose the English (US) keyboard layout.

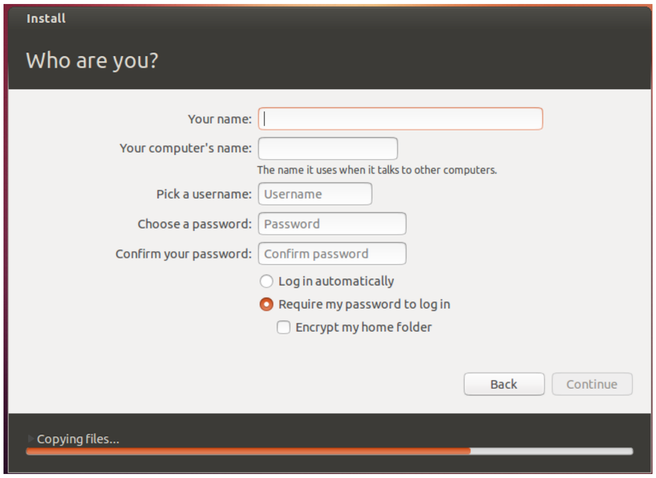

Figure 6 - Enter information about your virtual machine

h. Enter the information displayed in Figure 6, including your name, a name for your computer (use no spaces), a username (lowercase, no spaces), a password, and choose "Require my password to log in." Click "Continue."

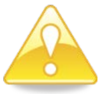

WARNING – Write down your username and password somewhere in case you forget it.

i. Be patient as Ubuntu finishes installing. When it is done, click “Restart Now.”
j. When prompted, press “Enter” to finish restarting the virtual machine.

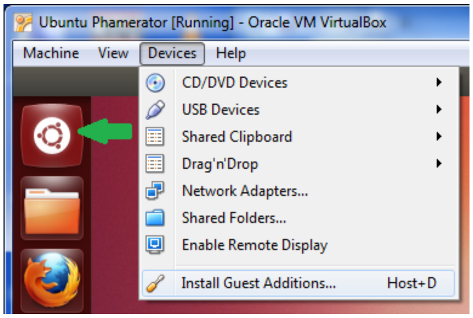

Figure 7 - Install Guest Additions

4. <u>Install the VirtualBox extensions package (Guest Additions)</u>
   a. With your virtual machine shut down, navigate to the universal extension pack and double click on it. Follow the directions to install it.
   b. Start your virtual machine by clicking the “Start” icon with the green arrow and log in.
   c. After you are logged in, click “Devices” at the top menu and choose “Install Guest Additions.” (See Figure 7)
   d. Click “Run” on the box that pops up. Enter your password in the next box and click “Authenticate” to install guest additions.
      The box with white text and a purple background that pops up is called Terminal. This is the window where most of the functions of setting up Phamerator will be performed.
   e. When the guest additions are finished installing the bottom line of text in the Terminal window will say “Press Return to close this window…” Press the enter key.

5. <u>Install Ubuntu Updates</u>
   a. Click on the “Dash” icon (indicated by the green arrow in Figure 7).
   b. Type “Software Updater” and click on it to run it.
   c. Install all available updates and restart Ubuntu when prompted.

6. <u>Learn to use basic Terminal commands</u>
   a. Navigate to the “Dash” icon (marked by the green arrow in Figure 7) and type in “Terminal.” Click and drag the “Terminal” icon and drop it underneath the Folder icon (shown in Figure 8 beneath the Dash Home icon).
   b. Click on the new Terminal icon to launch Terminal.
   c. To communicate with the computer through Terminal, you need to use a unique language. Terminal operates the same way you would operate your computer. Instead of clicking on things, you type commands into Terminal and press “Enter." Table 1 compares how you perform basic tasks using a mouse with how you perform those same tasks by typing commands in Terminal.

Table 1. Tasks to perform for setting up Phamerator in Terminal

**Task to Perform**	**How to do it on Windows or Mac OS**	**What you type in Linux (Terminal), then press “Enter.”**
Open a folder called “Documents”	Double click on “Documents.”	`cd Documents`<br>(cd means “Change Directory”)
Go up a folder level	Click the “Back” button	`cd ..`
Display the contents of a folder	Double click on the folder	`ls` (ls means list)
Start a program called Phamerator	Double click on the “Phamerator” icon	Within the folder containing “Phamerator,” type `./Phamerator` (./ means run)

IV. How to install Phamerator and other necessary programs

1. Use sudo apt-get to install software from the Internet

   a. To install programs you will enter a command in Terminal (see Figure 8).

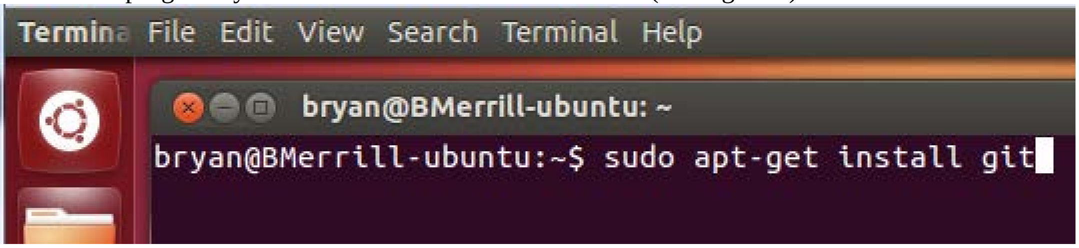

Figure 8 – Terminal window with command to install "git"

The "sudo" argument gives administrator privileges to the command that follows. The password you are prompted for is the password for the Ubuntu user account you are currently logged into (see step III-3-h on page 4 and Figure 6). Your account needs to be an Administrator account for this to work. (To check or change this, click in the top right corner, choose "System Preferences," and then "User Accounts.")

   b. Using this same procedure, install the following programs one at a time by typing the code below and press Enter. (For best results, install programs sequentially down the left column, and then down the right column.)

- `sudo apt-get install git`
- `sudo apt-get install python`
- `sudo apt-get install perl`
- `sudo apt-get install pyro`
- `sudo apt-get install clustalw`
- `sudo apt-get install clustalo`
- `sudo apt-get install curl`
- `sudo apt-get install perl-doc`
- `sudo apt-get install python-dev`
- `sudo apt-get install ncbi-blast+`
- `sudo apt-get install bzr`
- `sudo apt-get install python-pygoocanvas`
- `sudo apt-get install python-webkit`
- `sudo apt-get install python-mysqldb`
- `sudo apt-get install mysql-server` *
- `sudo apt-get install python-pip`
- `sudo pip install numpy`
- `sudo pip install biopython`
- `sudo apt-get install python-biopython`
- `sudo apt-get install python-pp`
- `sudo apt-get install blast2`

*** When installing mysql-server, you will be prompted to enter a root password. Don't forget this password!**

   c. While installing, you may have to type "`y`" (for yes) and then press enter.

   d. Outside Terminal, click on the Folder or Filing Cabinet icon (see Figure 8). This is your Home folder. Inside the Home folder, right-click and choose "Create new folder." Type "Applications" and press Enter.

   e. In Terminal, navigate to the "Applications" (`cd Applications`) folder and type the following command as it appears below. Then press Enter. This command will download Phamerator from a website and put a folder called "phamerator-dev" in your "Applications" folder.
   - `git clone https://github.com/byuphamerator/phamerator-dev.git`

   f. Change into the new "phamerator-dev" folder (`cd phamerator-dev`) and type the following command. Then press Enter.
   - `git pull`

   Each time you prepare to run Phamerator, you should download the latest updates of Phamerator by navigating to the "phamerator-dev" folder and running the "git pull" command.

2. Start Phamerator and download BLAST

   a. After running a `git pull`, type `cd phamerator` to navigate to the "phamerator" folder.

   b. Type `./Phamerator` and press enter through all of the prompts if there are no databases to load.

   c. Phamerator will start running, and ask you if you want to download BLAST. Allow it to do this.

   d. Congratulations! Phamerator is now ready to use.

   e. To run Phamerator, for the username type `root`, for the password use the same password you used when you installed MYSQL Server, for the server use `localhost` if running a database on your own computer, and for database use the name of the database you want to load. Notice that when Terminal is prompting you for your password it will look like nothing is happening as you type it. This is normal! Type it correctly anyways and press enter to bring up the prompt for the server and database name. If a popup box prompting you for your

password appears instead of the normal Phamerator window, it means that you typed your password incorrectly in the Terminal window. Click the X on the small popup window and try to start Phamerator again in Terminal.

IMPORTANT NOTE:
In command line, capital letters and lowercase letters are understood to be different. If something doesn't work, check to see if you have capital and lowercase letters in the correct places.

V. HOW TO BUILD A PHAMERATOR DATABASE

To build a Phamerator database containing phage genomes, you will first need to collect the files from GenBank and put them in a folder within your virtual machine. GenBank files can be generated and manipulated by DNA Master (http://cobamide2.bio.pitt.edu/computer.htm) or can they can be downloaded from NCBI (http://ncbi.nlm.nih.gov).

Since Phamerator uses GenBank files to build the phage database, the GenBank files need to include certain features. If the GenBank files are missing features, they will first need to be processed using DNA Master. Preparing GenBank files for Phamerator using DNA Master is a complicated process that requires some troubleshooting, so follow these instructions closely! Specific tips for issues that may arise are covered in section 6f.

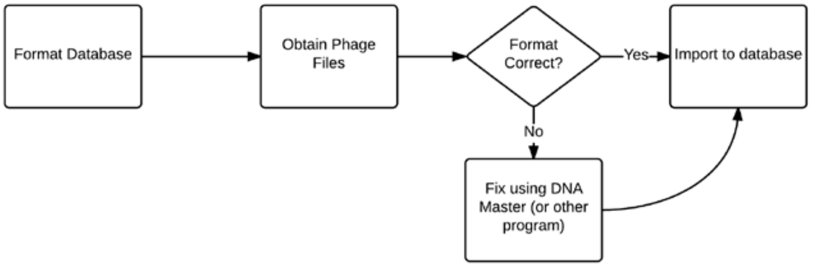

1. Prepare a new SQL database
   a. Create a new, blank MYSQL database using the following command in terminal, replacing "testphage" with the name you want for your database.
      - `mysql -u root -p -e 'create database testphage'`

      Press Enter. You will be prompted for a password, which is the password you created when you installed MYSQL Server (see step IV-1-b on page 6). This is the same password you will enter each time you are prompted throughout the rest of this documentation. `–u` stands for username, `-p` prompts for the password.
      Substitute "testphage" with what you want to name your database.
   b. If you want to create a MYSQL database on a networked MYSQL server (not on your computer), use this command:
      - `mysql -u root -p –h server.univ.edu -e 'create database testphage'`

      Substitute "server.univ.edu" with the address of the networked MYSQL server.
      The –h argument and the server name can be added to any of the commands described below for databases hosted on a server.
   c. Next we need to customize the blank database for use with Phamerator by first navigating in Terminal to the "sql" folder of the Phamerator directory. (`cd Applications/phamerator-dev/phamerator/sql/` from the home folder)
   d. Enter the following command: `mysql -u root -p testphage < db_schema.sql`
      Substitute "testphage" with the name of the database you created.
      GenBank files can now be imported into this database. Proceed to step 2.

To delete databases:

   e. Log in to MYSQL using the command `mysql –u root –p`, press Enter, type the password and press Enter. Enter the command: `drop database testphage;`
      Substitute "testphage" with the database you want to delete.

To export or back up a database into a single file:

   f. Use the following command.
      - `mysqldump –u root -p testphage --default_character_set=utf8 > "testphage.sql"`

      Replace "testphage" with the name of the database you want to export into a file, and "testphage.sql" with what you want to call the file. Just make sure it has a ".sql" at the end.

g. You can compress the SQL file so it can be emailed. Right-click on the SQL file and choose "Compress."

To import Phamerator databases from an SQL file:

h. Create a new database (step V-1-a on page 7)
i. Import the db_schema.sql file (step V-1-d on page 7)
j. In terminal, navigate to the folder where your dumped *.sql file is.
k. Enter the following command.
   - `mysql -u root -p testphage --default_character_set=utf8 < filename.sql`

   Substitute "testphage" with the name of the database you created and "filename.sql" with the name of the SQL file you are trying to import.

To show all MYSQL databases:

l. Enter the following command and press Enter.
   - `mysql -u root -p`
m. Enter the following command and press Enter.
   - `show databases;`

2. Collect GenBank files and determine if they are ready to import into Phamerator

a. Inside the virtual machine, download GenBank files from NCBI and rename each file with the correct phage name. If you did not save the files from NCBI in your Ubuntu virtual machine, you can email the files to yourself, use Dropbox, or click on the VirtualBox "Devices" menu option, go to "Drag'n'Drop" and choose "Bidirectional" which should allow you to drag files from one desktop to the other.
b. Examine each file to see if it is ready to be imported into Phamerator or if it will need modification. Figure 9 highlights features of the GenBank file that are important to Phamerator.

```
LOCUS       Jimmer1          54312 bp    DNA     linear   ENV 25-JAN-2013
DEFINITION  Jimmer1.
ACCESSION
VERSION
KEYWORDS    ENV.
SOURCE      Jimmer1
  ORGANISM  Jimmer1
            Unclassified.
REFERENCE   1  (bases 1 to 54312)
  AUTHORS   Merrill,B.D.
  TITLE     Direct Submission
  JOURNAL   Submitted (25-JAN-2013)
FEATURES             Location/Qualifiers
     source          1..54312
                     /organism="PL-Jimmer1"
                     /mol_type="genomic DNA"
                     /note="complete genome"
     gene            26..463
                     /gene="1"
                     /locus_tag="JIMMER1_1"
     CDS             26..463
                     /gene="1"
                     /locus_tag="JIMMER1_1"
                     /note="Terminase small subunit"
                     /codon_start=1
                     /transl_table=11
                     /product="gp1"
/translation="MKLTPKQQAFADYYIQIGNATEAARKAGYSDKTAKEVGYENLTK
PHIKAYIDERMAVKDAERIASQDEVLEFLTNVMRGKVTEKIPLGLGMGEQGLVKNELQ
GKDRIKAAELIGKRYGLWVEKVNLDGDLAVTIIDDIGVDDEEG"
```

Figure 9 - Example of a GenBank file containing features that are necessary for Phamerator

- **PL-Jimmer1** is the organism name. This is the name that will appear in Phamerator as the name of the phage. However, if there are any spaces between the quotation marks in this area, Phamerator will ***only*** import the last word after the last space and use that as the title of the phage.

IMPORTANT: As you prepare each GenBank file and modify the organism names as necessary, keep track of the phage names in an Excel spreadsheet with the names in the far left column.

- `Gene` and `CDS` are necessary for each gene, or protein described by the GenBank file. Occasionally, you will find GenBank files that do not contain the "gene" feature. If the "gene" feature is missing, then Phamerator will likely not import the file correctly. You should process the file with DNA Master first. The /gene="1", etc. is also critical.
- `/locus_tag="JIMMER1_1"` is one of the most important features in the GenBank file. If you cannot see a locus tag in the file, then you cannot import it into Phamerator. You will need to use DNA Master (or another program) to add phage-specific locus tags and then export a new GenBank file.
- `translation` – The first letter inside quotations after the "translation" section should be an "M" for each gene. If it is not, you may encounter an error in Terminal as you try to import the phage into Phamerator. When you export files from DNA Master, you must select "Bacterial and Plant Plastid Code." This results in `/transl_table=11` being displayed next to each gene. This helps the phage genome import correctly into Phamerator.

3. Using DNA Master to prepare GenBank files from NCBI for Phamerator

   If GenBank files from NCBI are missing any of the above features they need to be processed using DNA Master before they can be successfully imported into Phamerator. DNA Master only runs on Windows.

   a. Download DNA Master. It can be found at http://cobamide2.bio.pitt.edu/computer.htm. Make sure you have the most up-to-date version by running DNA Master as an Administrator (right click on the icon, choose "Run as Administrator"). After it starts choose "Help" and then "Update DNA Master."
   b. Paste the accession number for the phage you want to download into the bottom left corner of DNA Master. Click the red checkmark and then choose "Save."
   c. The /gene= feature of the GenBank file is populated by the information found in the "name" field in DNA Master and the /locus_tag= feature is populated from the Locus Tag field in DNA Master. You can automatically reassign gene numbers and locus tags by clicking "Validate". To reassign both, under the "Control" tab leave all boxes except "Label default products…" checked. Type your new locus tag prefix in the appropriate field. Under the "Numbering" tab, make sure both fields say "1". If you don't want to reassign locus tags, leave only "Assign Names…" checked.

   WARNING: Changing gene numbers so they are different than the original ones will make it difficult to compare gene numbers in Phamerator with gene numbers in the published genome.

   d. Occasionally phage genomes are not numbered sequentially. You may have to edit all of the name and locus tag fields manually if you wish to preserve the original gene numbers.
   e. Phages are often arranged in a manner that is inconsistent with other similar phages. If you wish to rearrange the phage genome so it begins at a new location, go to the top of DNA Master and choose "Genome", "Rearrange," and click on the "Rearrange" tab. Include all annotation except "Original Coordinates" and type in the base pair where you want the new genome to begin. Make sure this doesn't interrupt any genes and click "Rearrange."
   f. In the new window, uncheck "Assign new locus tags" as well as everything else on the right side of the window. Leave the checked features checked.
   g. Click "Parse."
   h. Save the DNA Master file.
   i. You can also reverse-complement the phage genome in a similar manner by choosing "DNA" at the top of the DNA Master window, click "Convert", and choose "Complement" and edit the "Parse box as above.
   j. If any of these steps do not produce the desired results, before rearranging or complementing click on the "documentation" tab and click "recreate documentation." You can also do this after rearranging or complementing.
   k. When your DNA Master file is ready, choose "File" and "Save as DNAM5 file" and save it to a specific location.

4. Using DNA Master to import FASTA files, auto-annotate them, and generate GenBank files for Phamerator

   a. In the top menu, choose "File", "Open", and choose "FastA Multiple Sequence File." Navigate to your FASTA file and click "Open."
   b. If you have multiple phage genomes in your FASTA file, click on the one you want to use. In the bottom right corner, choose "Export" and click "Create sequence from this entry only."
   c. In the DNA Master menu, choose "Genome" and then "Auto-Annotate." Under the "Tags & Comments" tab, change the Prefix to the phage name or whatever you want the locus tag prefix to be. Click "Annotate" and save the resulting DNA Master file.

5. Using DNA Master to create a GenBank file
    a. Before beginning this process, you can choose to hide text in the “notes” box for each gene. To hide all of these from the resulting GenBank file, click on the small drop-down arrow and choose “Privatize all.” Everything inside the <private> and </private> designation will not appear in the /notes= section of the GenBank file.
    b. To create the GenBank file, click on the “Tools” menu in DNA Master and choose “Submit to GenBank.”
    c. Click “Add” and navigate to the DNAM5 file you have saved.
    d. Under the “Description” tab, the Locus field needs to be no longer than eight characters. It is usually the accession number. Paste this into the “Accession” field as well.
    e. The Genetic Code field should be Bacteria and Plant Plastid code.
    f. Under the “Organism” tab, fill in the “Organism” field with the phage name (remember, Phamerator only imports the last word of whatever is in this field if it contains any spaces) and the Lab Host with the host of the phage.
    g. Under the “References” tab, click “Add.”
    h. Under the “Process” tab, check “Export Flat File”, “Include ‘gene’ features”, “Include locus tags”, and “Restrict to a single reference.”
    i. Click “Process single project” and save the resulting GenBank file as (phage_name).gb.fixed or choose “Process all listed projects” and choose a folder where GenBank files for all phages ready for processing will be created. All GenBank files to be imported into Phamerator need to end in “.fixed”.

6. Import GenBank “fixed” files into Phamerator

A good method for building a Phamerator database is importing one GenBank file at a time into a “test” Phamerator database. If it works, move the GenBank file to a folder for correctly formatted files. If it doesn’t work, move it to a folder for incorrectly formatted files. You’ll then need to troubleshoot it and make a new file using DNA Master. Before you try to import it again, you’ll need to delete the phage from the test database (step V-6-j on page 11) or delete and recreate the database (step V-1-e on page 7).

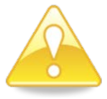

WARNING – Once you import a sequence into Phamerator, sometimes you cannot delete it without deleting everything in the database. Therefore, if you encounter any of the errors described below, be sure you really want to import the sequence.

    a. Click on the Folder icon and navigate to the “phamerator-dev” folder. Inside the “phamerator-dev” folder, create a folder called “importseq,” one called “importseqworked” and one called “importseqfail.” You will transfer GenBank files one at a time to the “importseq” folder to import into Phamerator. If it works, it goes to the “importseqworked” folder; if it doesn’t, move it to the “importseqfail” folder.
    b. Navigate to the folder where your GenBank files are located. The file name will be something like Jimmer1.gb (or Jimmer1.gbf). Right click on each file and change the file to Jimmer1.gb.**fixed**. Transfer one of the *.gb.fixed files to the “importseq” folder.
    c. Open Terminal. Navigate to the “phamerator” directory. This can be done by entering the command:
        - `cd Applications/phamerator-dev/phamerator`
    d. Run the import sequence script by entering the following command and pressing Enter. Phamerator will ask you whether you want to import all the contents of the folder, so type “y” for yes and press Enter. Replace testphage with the name of the database you want to receive the GenBank files.
        - `./phamerator_manage_db.py -u root -p -s localhost -d testphage -i ../importseq/`
          You will be prompted for your MYSQL server password (see step IV-1-b on page 6).
          This is what each part of this command means:
        - `phamerator_manage_db.py` is the program used to import the sequences
        - `-u` tells Terminal the next thing you type is the username. `root` is the username
        - `-p` tells Terminal to ask for your password
        - `-s` tells Terminal the next thing you type is the server address Use `localhost` if running a local database.
        - `-d` tells Terminal the next thing you type is the database name. `testphage` is the example database name
        - `-i` tells Terminal the next thing you type is the location of the sequence to import. `../importseq/` tells Terminal to go down one directory import all of the GenBank files in the folder named “importseq”.
    e. Phamerator will warn you that all phages in the directory you specified (“importseq”) will be imported. Type “Y” and press Enter to continue.
    f. There are a few errors you may encounter. If you encounter an error, Terminal will ask you whether or not you want to proceed. Type “**n**” for no, and press enter and the database should remain unchanged. If a phage

genome is imported and the Phamerator user interface indicates that the genome has zero genes, delete the phage and try again (step V-6-j below). Common errors include:

- Invalid start codon (usually not an issue, just press "Y" and continue importing)
- Translation from GenBank file does not match translation computed by Phamerator (usually not an issue, just press "Y" and continue importing). This is caused when the translation table is something other than "Bacterial and Plant Plastid" or /transl_table=11.
- GeneID already in use (the locus tag for this gene is not unique among all genes in the database). Exit and generate new unique locus tags for this genome using DNA Master
- GeneID truncated (your organism name and locus tag are too long). Shorten one or both using Find and Replace in the Ubuntu text editor.

g. After the import process is completed, you will receive a prompt asking you whether you want to delete the ClustalW and BLAST scores. Type "N" for no and press Enter.

h. To see if the GenBank file imported correctly, type `./Phamerator` and press Enter. Type in your username (`root`), password (your mysql server password), the server address (`localhost`), and the database name as prompted by Terminal. If a popup window appears asking you for your password again, you typed it wrong. Click the red "X" and start Phamerator again.

i. After Phamerator starts, click on the "Phages" tab on the left. If all of the information is correct, move the GenBank file from "importseq" to either the "worked" or "failed" folder. Move another GenBank file into the "importseq" folder, and go to the Terminal window. By pressing the up arrow on your keyboard, the previous command you used is filled in and you can simply press Enter to import the next file.

Deleting phage genomes from a Phamerator database:

j. You can use the `phamerator_manage_db.py` script to delete phage entries from the database that did not import correctly. You will have to start Phamerator to make sure it was successfully removed.

- `./phamerator_manage_db.py -u root -p -s localhost -d testphage --remove name:TA17A`

Substitute "testphage" for your database name and "TA17A" for the name of the phage you are deleting. Type it exactly as it appeared in the "Phages" tab in Phamerator.

WARNING – When you remove phages a message will tell you that doing so invalidates phage protein phamily assignments and prompts you to either erase them "yes" or leave them alone "no." If you want to preserve the existing pham numbers, always choose "no." You can re-run phamBuilder4 later and it will update and not renumber the phams from scratch.

k. After you have one folder containing all of the fixed GenBank files that have been successfully imported into Phamerator using your test database, create a new database and import these files (step V-6-d on page 10).

7. Adding cluster designations to the Phamerator database

a. Cluster designations can be used to group phages based on the host they infect, phage morphology, or other user-defined characteristics. In the spreadsheet you made of each phage name (Section V-2-b), assign a cluster in the cell immediately to the right of each phage. Cluster names can be up to 5 characters long and contain only letters and numbers. Phage that are "singletons" that don't have a cluster assignment should have "NON" as their cluster. (You will need at least one phage in the NON cluster or your pham circles will not display).

b. The table will look like this:

Jimmer1	A
Jimmer2	A
Emery	B

c. To generate the cluster file you will need to import into Phamerator, select the two-column table from top to bottom and copy it. Paste it into Notepad or a basic text editor. Click "File, Save As…" and type a file name. Save it as a *.csv file. Place it inside a folder named "cluster" that you create inside the "phamerator-dev" directory.

d. In Terminal, navigate to /phamerator-dev/phamerator/plugins and perform the following command.

- `./update_cluster.py -u root -p -s localhost -d testphage --update_cluster ../../cluster/phage_cluster.csv`

Substitute "testsphage" with the name of the database you are using and phage_cluster.csv with the name of your cluster file.

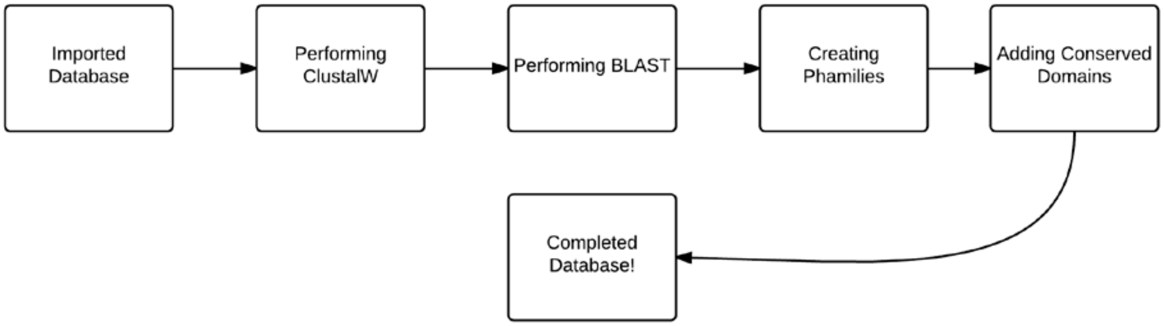

8. Starting Phamerator

After adding phages or clusters to the Phamerator database but before performing computations, you can start Phamerator to make sure the phages imported correctly. You will only be able to access the data in the "phages" tab until all computation steps are completed.

a. To start Phamerator, navigate (in Terminal) to Applications/phamerator-dev/phamerator.
b. Type `./Phamerator`. You will be prompted for username, password, server, and database name.
c. If you are running a local database, you can use "`root`" for username, the MYSQL server password, "`localhost`" for server, and type the name of the database you want to load for "`database`".
d. If a popup window appears prompting you to re-enter the password, you typed incorrectly in the Terminal window. Click on the red "X" and start Phamerator again.

9. Perform ClustalW computations on the Phamerator database

These computations require a lot of computing power and may take a very long time. Be prepared for this. ClustalW and BLAST are run using a server-client interaction. One Terminal window (the server) will look through the database and generate jobs that it will feed to the other window (the client). The client Terminal window will perform computations and feed the back to the server Terminal window, which inserts any significant results into the Phamerator database. Each "job" compares one gene product to every other gene product in the database.

a. In terminal, navigate to the "Applications/phamerator-dev/phamerator" directory. Type the following command and press Enter. The number following `–i` indicates how many instances of the client you want to run. Choose `1` for most computers, or `2` for a very fast computer. Make sure the `–l True` is actually a lowercase L. *Substitute "testphage" for your database.*

- `./phamServer_InnoDB.py -u root -p -s localhost -n localhost -d testphage -i 1 -l True -a clustalw`

b. Open a second Terminal window by right clicking on the Terminal icon and choose "New Terminal Window". Navigate to the "Applications/phamerator-dev/phamerator" directory, type the following command and press Enter.

- `./phamClientOmega.py -u root -p -n localhost`

If you specified that you want to run two instances, open a third Terminal window, navigate to the "Applications/phamerator-dev/phamerator" directory and enter the above command.

WARNING – This process can take a **VERY** long time. If you need to pause the process and resume a different time, press Ctrl + C on your keyboard in each *client* Terminal window first, followed by the server window and the process will stop. When you start it again, it will pick up where it left off.

c. When this long process is completed, you will notice that the first window repeatedly displays "No work units available… Sleeping…" You can safely end the processes in each window using Ctrl + C and move on to the next step.

10. Perform BLAST computations on the Phamerator database

a. The second process that needs to be run is BLAST. This process also requires multiple terminal windows (which can be opened by right-clicking on the Terminal icon and choosing New Terminal). This process also requires some setup. Note that this process does NOT link to the internet or use the BLAST feature found on NCBI. It runs locally, comparing only the data found in the database. You can run as many instances of BLAST as you have processor cores assigned to the virtual machine. For each instance, you will need to create separate folders in the "phamerator-dev" folder for BLAST data. Name them blastdata, blastdata2, blastdata3, etc. and make as many as you have cores on the computer. From within the "phamerator" directory, type the following

command in one Terminal window and press Enter. To change the number of instances, substitute that number for “4” in the following command.
   - `./phamServer_InnoDB.py -u root -p -s localhost -n localhost -d testphage -i 4 -l True -a blast`

b. In the second window, navigate to “Applications/phamerator-dev/phamerator”, type the following command and press Enter.
   - `./blastclient.py -u root -p -n localhost -a ../../BLAST/bin -d ../blastdata/`

c. Open a third, fourth, and fifth window, navigate to the correct directory, and use the exact same command as above except for the final argument (`../blastdata/`). In the third window, change `../blastdata/` to `../blastdata2/,` in the fourth window to `../blastdata3/`, etc.

d. This process will also tell you when it is done. When it says “Sleeping…” press Ctrl + C to end it.

11. <u>Check the progress of ClustalW or BLAST</u>
Since ClustalW and BLAST can take a long time complete you can open an additional Terminal window and run a script to display the progress. To show the ClustalW or BLAST progress meter, do the following.
   a. Navigate to “Applications/phamerator-dev/phamerator”.
   b. Run the phamServer_progress.py script by using the following command.
      - `./phamServer_progress.py –u root –p –s localhost –d testphage –r 10 –a clustalw`
   c. Replace `testphage` with the name of your database and `–a` with `blast` if you are running BLAST.
   d. The number after the `–r` argument indicates how often (in seconds) you want to refresh the progress bar. The current command will refresh it every 10 seconds. Click “Cancel” to close the progress bar window. This will NOT affect ClustalW or BLAST processing. You can close and reopen this window as you wish by running the script above.
   e. When ClustalW or BLAST is finished processing, the progress window will automatically disappear and Terminal will display “(clustalw or blast) processing is complete”. Also, the Terminal windows running BLAST or ClustalW will say “No work units available…. Sleeping…”

12. <u>Assign phamilies (phams) for gene products in the Phamerator database</u>
   a. Assign phamilies using the data that ClustalW and BLAST have computed. Type the following command in Terminal from the “phamerator” directory and press Enter.
      - `./phamBuilder4.py -u root -p -s localhost -d testphage -c 0.325 -b 1e-50`
   b. The default parameters for phamBuilder are 32.5% identity and an E-value of 1e-50. Phamilies are created when a gene product has more than 32.5% identity or an E-value of 1e-50 with another gene product. You can modify these parameters to by using the same format and substituting different numbers for 0.325 and 1e-50.
   c. When this process is complete, your Phamerator phage database is ready to use!!!
   d. If you haven’t assigned clusters yet, you can do that now (see V-7). Databases are still functional even if no clusters are assigned.

13. <u>Setting up a local copy of the Conserved Domain Database (CDD)</u>
If the Conserved Domain Database (CDD) has not been set up on your computer, you will need to do the following steps. They are also rather time-consuming and need lots of processing power, but you only have to do it once. Make sure that you have <u>*at least*</u> 40 GB free on your Ubuntu virtual hard drive.
   a. Go to NCBI's CDD website (ftp://ftp.ncbi.nih.gov/pub/mmdb/cdd/)
   b. Download the file “**cdd.tar.gz**” (this needs to be done inside Ubuntu, or else the file needs to be moved there later.) It is a very large file (~3 GB).
   c. Place this newly downloaded file inside the phamerator-dev directory
   d. Make a new folder called cdd_db
   e. Open terminal
   f. Navigate to Applications/phamerator-dev and run the following command:
      `tar -xvzf cdd.tar.gz -C cdd_db/`
      You’ll see a ton of lines of code fly by, and will know when it’s done when a fresh command line appears.
   g. In terminal, navigate to Applications/phamerator-dev/cdd_db
   h. Type the first command listed below to set up the CDD. Press enter. When it is done, type the second command, press enter, and wait until it is done. Do this for all six.

makeprofiledb -title SMART.v6.0 -in Smart.pn -out Smart -threshold 9.82 -scale 100.0 -dbtype rps -index true
makeprofiledb -title Pfam.v.26.0 -in Pfam.pn -out Pfam -threshold 9.82 -scale 100.0 -dbtype rps -index true
makeprofiledb -title COG.v.1.0 -in Cog.pn -out Cog -threshold 9.82 -scale 100.0 -dbtype rps -index true
makeprofiledb -title KOG.v.1.0 -in Kog.pn -out Kog -threshold 9.82 -scale 100.0 -dbtype rps -index true
makeprofiledb -title CDD.v.3.10 -in Cdd.pn -out Cdd -threshold 9.82 -scale 100.0 -dbtype rps -index true
makeprofiledb -title PRK.v.6.00 -in Prk.pn -out Prk -threshold 9.82 -scale 100.0 -dbtype rps -index true

i. You're done creating the local CDD! Now you can run the cddSearch.py script and identify conserved domains in all of the genes in your Phamerator database.

14. Using the Conserved Domain Database (CDD) Search
    a. To identify conserved domains in each gene product in the Phamerator database, run the following command in Terminal from the "Applications/phamerator-dev/phamerator/plugins" directory. You will then be prompted for the username, password, server, and database.
       - `./cddSearch.py ../../cdd_db/Cdd ../../cddfasta/query.fasta`
    b. This command can be run at the same time as BLAST or ClustalW. (ClustalW and BLAST cannot be run at the same time.) Please be aware that aside from what happens immediately after you start this script, you will NOT see anything happen in the Terminal window until the process is completely done. Just let it process until you see a new blank line appear. This could take a few hours.

15. Modifying the Phamerator Database
    a. You can add additional phages or remove phages in the Phamerator database without reassigning all of the phams or deleting the BLAST and ClustalW scores. When adding or removing phages, you will be prompted to delete all BLAST or ClustalW scores or to leave them alone. If you don't want to recompute all of these scores, make sure you don't delete them (specify "N" for no)! Also, if you want to keep the phams, make sure you do not delete pham assignments when prompted (even though Phamerator says that removing phages invalidates pham assignments). You can always re-run phamBuilder4 to fix existing phams rather than delete the phams and build them from scratch.
    b. After you are finished modifying your database, re-run ClustalW, BLAST, phamBuilder4, and cddSearch.
    c. Your modified database is ready to use.

VI. HOW TO USE THE PHAMERATOR USER INTERFACE TO EXPLORE THE DATABASE

1. Displaying linear genome maps
    a. Start Phamerator by typing `./Phamerator` within the "Applications/phamerator-dev/phamerator" directory and enter your username, password, server address, and database name.
    b. Phamerator will start. Click on the "Phages" tab.
    c. While holding the Ctrl button down, click on all of the phages you want to appear in the map.
    d. Click the "Map" button at the top of the screen. You will see lots of code fly by in Terminal, and eventually a new window will open displaying the genome map. You can zoom in and out or reorder the map by clicking on the phage name and dragging it up or down.
    e. You can align the maps by clicking on one gene in each genome and choosing "align left" or "align right".
    f. You can save this map by clicking "File" then "Save As…" and choose a PDF file.
    g. Figure 10 below shows what a linear genome map of five phages looks like.

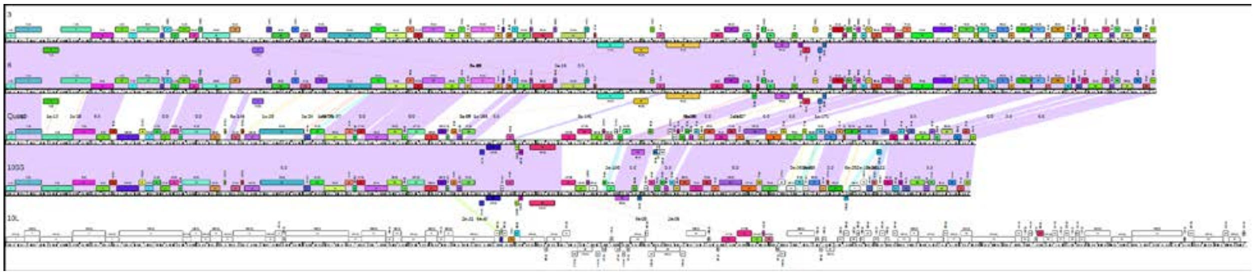

Figure 10 - Linear Genome Map for Five Phages

2. Displaying phamily circles
    a. Click on the “Phams” tab.
    b. Click on one pham in the list
    c. Click on the “Phamily Circle” button. When the window appears, you can zoom in and out and save the file as a PDF.
    d. Blue connecting lines represent linkage by more than 32.5% ClustalW similarity, and red connecting lines represent linkage by BLAST e-values less than 1e-50.

3. Exporting Pham and Cluster Tables
    a. Click on the Phages tab.
    b. Go to File > Export Pham Table. This is a csv file which can be imported into Excel using Data > Import from Text and then finding that file. With a little modification, it is a very powerful tool. If the conserved domains have not been added using cddSearch, then all cells in the “domains” column will say “None.” If cddSearch has been run, then any cells in the “domains” column that say “None” have no identifiable conserved domains.

4. Exporting genomes, genes, or proteomes
    a. Select phages in the “Phages” tab. Click “File” and choose whether to export a single FASTA file containing all of the genomes, genes, or proteins belonging to the phages selected.
    b. Select a pham in the “Phams” tab. Click “File” and choose whether to export a single FASTA file containing the gene or protein sequences for the members of the pham.

5. Accessing the Phamerator Quick Start User Manual
    a. For a description about other things you can do with Phamerator, go to Help and click on “User Manual.”

VII. CONCLUSION

Congratulations! Your Phamerator database is now functional. You have now learned how to:

- Create a Ubuntu virtual machine
- Install Phamerator and the software it needs to run
- Create a new Phamerator phage database by importing fixed GenBank files, processing them using ClustalW and BLAST, assign phamilies, and identify conserved domains
- Operate the Phamerator graphical user interface

You are now ready to use your knowledge of phage genomics and molecular biology to begin an in-depth analysis of these phages.

VIII. TROUBLESHOOTING AND FREQUENTLY ASKED QUESTIONS

For errors and problems that you are unable to fix, contact Bryan Merrill (brymerr921@gmail.com), Andy Ward (andytward9@gmail.com), Scott Carlson (scocarl@ls.byu.edu), or Dr. Sandra Burnett (sandra.h.burnett@gmail.com).

When starting Phamerator, a popup box appeared instead of the main Phamerator window.
- Password was typed incorrectly. Click the red X to close the popup window and start Phamerator again.

When I imported a phage into the database, the name is not displayed correctly in Phamerator.
- If there are spaces in the "/organism=" field of the GenBank file, only the last word is imported as the phage name. Replace spaces with dashes or underscores.

While trying to import phages, I get an error such as duplicated gene IDs.
- See section V-6-f on page 10.

I can't remember the names of all of the Phamerator databases I have created.
- See section V-1-m on page 8

Phamily circles will not display.
- No phages are assigned as "singletons." Assign a phage as a "singleton" and try again. See section V-7-a on page 11.

IX. APPENDIX OF PHAMERATOR TERMINAL COMMANDS

Install Phamerator dependencies (can be done in any directory) – IV-1-b, on page 6

- `sudo apt-get install git`
- `sudo apt-get install python`
- `sudo apt-get install perl`
- `sudo apt-get install pyro`
- `sudo apt-get install clustalw`
- `sudo apt-get install clustalo`
- `sudo apt-get install curl`
- `sudo apt-get install perl-doc`
- `sudo apt-get install python-dev`
- `sudo apt-get install ncbi-blast+`
- `sudo apt-get install bzr`
- `sudo apt-get install python-pygoocanvas`
- `sudo apt-get install python-webkit`
- `sudo apt-get install python-mysqldb`
- `sudo apt-get install mysql-server`
- `sudo apt-get install python-pip`
- `sudo pip install numpy`
- `sudo pip install biopython`
- `sudo apt-get install python-biopython`
- `sudo apt-get install python-pp`
- `sudo apt-get install blast2`

Install Phamerator (run in "Applications" directory) – IV-1-e, son page 6

- `git clone https://github.com/byuphamerator/phamerator-dev.git`

Update Phamerator (run in "Applications/phamerator-dev" directory) – IV-1-f, on page 6

- `git pull`

To start Phamerator (run in "Applications/phamerator-dev/phamerator/" directory) - IV-2-e, on page 6

- `./Phamerator`. You will be prompted for username (`root`), password (MYSQL server password), server (`localhost`), and database name.
- If a popup window appears prompting you to re-enter the password, you typed incorrectly in the Terminal window. Click on the red "X" and start Phamerator again.

Create new MYSQL database (can be done in any directory) – V-1-a, on page 7

- `mysql -u root -p -e 'create database testphage'`

Prepare MYSQL database for Phamerator use (run in "Applications/phamerator-dev/phamerator/sql" directory)
V-1-d, on page 7

- `mysql -u root -p testphage < db_schema.sql`

Delete MSQL database (can be done in any directory) – V-1-e, on page 7

- `mysql –u root –p`, press Enter, type the password and press Enter.
- `drop database testphage;` and press Enter

Export MYSQL database to single file (run this command in the directory where you want the file to end up)
V-1-f, on page 7

- `mysqldump –u root -p testphage --default_character_set=utf8 > "testphage.sql"`

Compress SQL file – V-1-g, on page 8

- Right click on SQL file and choose "Compress."

Import saved SQL file into new database – V-1-k, on page 8

- Create new MYSQL database
- Prepare MYSQL database for Phamerator use
- From directory where SQL file to import is stored, run:
  - `mysql -u root -p testphage --default_character_set=utf8 < filename.sql`

Show all MYSQL databases – V-1-m, on page 8

Enter the following command and press Enter.

- `mysql -u root –p`

Enter the following command and press Enter.

- `show databases;`

Import GenBank files into Phamerator database (run in “Applications/phamerator-dev/phamerator/” directory)
V-6-d, on page 10

- ./phamerator_manage_db.py -u root -p -s localhost -d testphage -i ../importseq/

Remove genomes from Phamerator database (run in “Applications/phamerator-dev/phamerator/” directory)
V-6-j, on page 11

- ./phamerator_manage_db.py -u root -p -s localhost -d testphage --remove name:TA17A
-

Add cluster designations to phages in Phamerator database (run in “Applications/phamerator-dev/phamerator/plugins” directory) – V-7-d, on page 11

- ./update_cluster.py -u root -p -s localhost -d testphage --update_cluster ../../cluster/phage_cluster.csv

Start server for processing ClustalW jobs (run in “Applications/phamerator-dev/phamerator/” directory)
V-9-a, on page 12

- ./phamServer_InnoDB.py -u root -p -s localhost -n localhost -d testphage -i 1 -l True -a clustalw

Start client for processing ClustalW jobs (run in “Applications/phamerator-dev/phamerator/” directory)
V-9-b, on page 12

- ./phamClientOmega.py -u root -p -n localhost

Start server for processing BLAST jobs (run in “Applications/phamerator-dev/phamerator/” directory)
V-10-a, on page 12

- ./phamServer_InnoDB.py -u root -p -s localhost -n localhost -d testphage -i 4 -l True -a blast

Start client for processing BLAST jobs (run in “Applications/phamerator-dev/phamerator/” directory)
V-10-b, on page 13

- ./blastclient.py -u root -p -n localhost -a ../../BLAST/bin -d ../blastdata/

Start progress window for ClustalW or BLAST jobs (run in “Applications/phamerator-dev/phamerator/” directory)
V-11-b, on page 13

- ./phamServer_progress.py –u root –p –s localhost –d testphage –r 10 –a clustalw

Build phamilies
V-12-a, on page 13

- ./phamBuilder4.py -u root -p -s localhost -d testphage -c 0.325 -b 1e-50

After downloading the cdd.tar.gz file (conserved domain database), extract the contents (run in “Applications/phamerator-dev/” directory) – V-13-f, on page 13

- tar -xvzf cdd.tar.gz -C cdd_db/

Build CDD database (run in “Applications/phamerator-dev/cdd_db/” directory) – V-13-h, on page 13

makeprofiledb -title SMART.v6.0 -in Smart.pn -out Smart -threshold 9.82 -scale 100.0 -dbtype rps -index true
makeprofiledb -title Pfam.v.26.0 -in Pfam.pn -out Pfam -threshold 9.82 -scale 100.0 -dbtype rps -index true
makeprofiledb -title COG.v.1.0 -in Cog.pn -out Cog -threshold 9.82 -scale 100.0 -dbtype rps -index true
makeprofiledb -title KOG.v.1.0 -in Kog.pn -out Kog -threshold 9.82 -scale 100.0 -dbtype rps -index true
makeprofiledb -title CDD.v.3.10 -in Cdd.pn -out Cdd -threshold 9.82 -scale 100.0 -dbtype rps -index true
makeprofiledb -title PRK.v.6.00 -in Prk.pn -out Prk -threshold 9.82 -scale 100.0 -dbtype rps -index true

Add conserved domains to gene products in Phamerator database (run in “Applications/phamerator-dev/phamerator/plugins/” directory) – V-14-a, on page 14

- ./cddSearch.py ../../cdd_db/Cdd ../../cddfasta/query.fasta
